# A Robust and Rapid Candidate Gene Mapping Pipeline Based on M2 Populations

**DOI:** 10.3389/fpls.2021.681816

**Published:** 2021-06-02

**Authors:** Huangkai Zhou, Kuanqiang Tang, Guang Li, Wenqiang Liu, Hui Yu, Xiaohui Yuan, Suxin Yang, Madan K. Bhattacharyya, Xianzhong Feng

**Affiliations:** ^1^Key Laboratory of Soybean Molecular Design Breeding, Northeast Institute of Geography and Agroecology, The Innovative Academy of Seed Design, Chinese Academy of Sciences, Changchun, China; ^2^College of Advanced Agricultural Sciences, University of Chinese Academy of Sciences, Beijing, China; ^3^Guangzhou Gene Denovo Biotechnology Co. Ltd, Guangzhou, China; ^4^School of Computer Science and Technology, Wuhan University of Technology, Wuhan, China; ^5^Department of Agronomy, Iowa State University, Ames, IA, United States

**Keywords:** bulked segregant analysis, whole-genome sequencing, mutagenic variants, M2 generation, functional gene mapping

## Abstract

The whole-genome sequencing-based bulked segregant analysis (WGS-BSA) has facilitated the mapping candidate causal variations for cloning target plant genes. Here, we report an improved WGS-BSA method termed as M2-seq to expedite the mapping candidate mutant loci by studying just M_2_ generation. It is an efficient mutant gene mapping tool, rapid, and comparable to the previously reported approaches, such as Mutmap and Mutmap+ that require studying M_3_ or advanced selfed generations. In M2-seq, background variations among the M_2_ populations can be removed efficiently without knowledge of the variations of the wild-type progenitor plant. Furthermore, the use of absolute delta single-nucleotide polymorphism (SNP) index values can effectively remove the background variation caused by repulsion phase linkages of adjacent mutant alleles; and thereby facilitating the identification of the causal mutation in target genes. Here, we demonstrated the application of M2-seq in successfully mapping the genomic regions harboring causal mutations for mutant phenotypes among 10 independent M_2_ populations of soybean. The mapping candidate mutant genes just in M_2_ generation with the aid of the M2-seq method should be particularly useful in expediting gene cloning especially among the plant species with long generation time.

## Introduction

With the development of next-generation sequencing (NGS) and a continual drop in the cost of whole-genome sequencing-based bulked segregant analysis (WGS-BSA) has become a routine tool for rapid mapping of candidate genes. At present, various WGS-based BSA methods have been developed for identifying qualitative or quantitative loci (QTL) with large effects. For example, QTL-seq (Takagi et al., 2013) is an efficient method for QTL mapping using F_2_ or recombinant inbred lines (RILs) developed by hybridizing distantly related varieties. However, a large number of candidate functional variations were detected within the candidate region by using WGS-based BSA methods. To finally identify the key causal mutation, a large segregating population is required for fine mapping.

To avoid dependency on time-consuming fine mapping, the study of a segregating mutant population is an effective alternative strategy. Only a limited number of mutant genotypes, as opposed to thousands of segregant individuals in fine mapping, were required in mutant-based strategies. Mutmap (Abe et al., 2012) is a representative method that is mainly applied for mapping the point mutations induced by chemical mutagen, ethyl methanesulfonate (EMS). As EMS mutagenesis can generate thousands of random mutations across the genome, EMS-induced mutations could be used as markers in BSA mapping. The mutation density between mutant and wild-type lines is usually sparse, not more than 5–10 mutations/Mb, of which only a limited number is mapped to the target genomic regions. Thus, it is beneficial to determine the causal mutation directly by using WGS-based BSA methods (Schneeberger et al., 2009; Abe et al., 2012; Fekih et al., 2013). However, before hybridizing the mutants to the wild-type line in the protocol of Mutmap method, mutants are often selfing several generations, to ensure that they are controlled by recessive causal mutation, and to obtain the mutant with homozygous mutation. The Mutmap method has three potential limitations. First, not all mutants are suitable for selfing to obtain homozygous progenies. For example, some recessive mutations may cause early development lethality or sterility and they only can be maintained in heterozygous condition. Second, at least two generations of selfing of the mutants are required prior to hybridizing to the wild-type lines. Third, it requires the hybridization of mutants with wild-type lines for raising segregating materials, which is time consuming and labor intensive. Thus, Mutmap is a very time-consuming method and it has limited use especially in the species with long generation time. The first and third shortcomings have been resolved by Mutmap+ (Fekih et al., 2013). In this method, M_2_ plants harboring early development lethality recessive mutations in heterozygous condition are selfing to generate the M_3_ population. Segregating sub-M_3_ populations is used to map the causal mutations by applying the BSA strategy. Nonetheless, the second shortcoming has not yet been completely resolved in Mutmap+, and it also requires at least two generations of selfing to raise the M_3_ generation.

In this study, we developed an improved WGS-based BSA method termed as M2-seq that does not require selfing of M_2_ to raise the M_3_ generation as in Mutmap+. Individuals with a mutant phenotype in M_2_ generation are identified and pooled to obtain the mutant bulk. Similarly, a wild-type bulk is created by using wild-type M_2_ progenies. We have validated this approach by mapping the genomic regions harboring causal mutations among 10 soybean mutants. The potential interference factors of M2-seq, such as residual background variations and genetic chimeras, are discussed.

## Materials and Methods

### Plant Material

The soybean (*Glycine max*) mutants used in this study were generated by EMS for the treatment of seeds of a cultivar “IGA 1008,” which was derived from *Williams 82*. Seeds of IGA1008 were immersed in 0.6% EMS (Sigma-Aldrich, Saint Louis, MO, United States) solution in an airtight container for 6 h. The container was placed in a fume hood and shaken evenly every half an hour. Then, the seeds were rinsed three times with 0.1 M sodium thiosulfate. Finally, the seeds were washed under running tap water for 40 min, and stored after being dried in a fume hood. The EMS waste liquid was neutralized with equal volume of 1 M sodium thiosulfate, then sent to institute chemical treatment station. All the above experimental operation procedures conform to the standard biosafety and institutional safety procedures. The mutagenized M_1_ seeds were grown in the Chang-Chun experiment field of Northeast Institute of Geography and Agroecology. M_1_ EMS mutants of soybean were self-pollinated for the generation of M_2_. For the identification of the candidate mutants with the aid of M2-seq application, the number of wild-type and mutant progeny in each M_2_ population was calculated. Chi-square was applied to verify the ratio of individuals with wild-type and mutant phenotypes in M2, which deviated from 3:1 and indicated the standard recessive mutant. In each of the M_2_ populations, leaves of 15 or more progenies with either wild-type or mutant phenotype were collected in equal proportions and used to prepare DNA. Thus, 20 DNA samples were prepared for example from 10 M_2_ populations.

### Whole-Genome Sequence of Bulked DNA

Genomic DNA was extracted from each bulked leaf sample by using the Plant Genomic DNA kit (Tiangen, Beijing, China) for sequencing. Libraries with 350-bp inserted fragments were constructed by using a TruSeq Nano DNA HT Sample Preparation Kit (Illumina Inc., San Diego, CA, United States) according to the manufacturer’s protocol and sequenced by using an Illumina HiSeqX instrument to obtain 150-bp paired-end reads. The sequences are available from The National Center for Biotechnology Information (NCBI) database with SRA number SRP191330.

### Reads’ Alignment and Single-Nucleotide Polymorphism Calling

Quality trimming is an essential step to generate high confidence data in variant calling. Raw reads were processed to obtain high-quality clean reads according to four stringent filtering standards: (1) removed reads with≥10% unidentified nucleotides; (2) removed reads with>50% bases with Phred quality scores of ≤20; and (3) removed reads aligned to the barcode adapter. To identify single-nucleotide polymorphisms (SNPs) and indels, Burrows–Wheeler Aligner (BWA, v0.7.16a) was used to align the clean reads from each sample against the soybean reference genome (*G. max* Wm82.a2.v1) with the settings “mem 4 k 32 M,” where k is the minimum read length and M is used to mark the short split alignment hits as secondary alignments. Variant calling was performed for all samples by using the GATK (v3.8) Unified Genotyper program. SNPs and indels were filtered by using GATK Variant Filtration with appropriate standards (Window 4, filter “QD < 4.0 || FS > 60.0 || MQ < 40.0,” G_filter “GQ < 20”), and those exhibiting segregation distortion or sequencing errors were discarded. In order to determine the physical positions of each SNP, the software tool ANNOVAR (Wang et al., 2010) was used to align and annotate the SNPs or indels. Subsequently, SNPs were used to construct a phylogenetic tree by a neighbor-joining method using PHYLIP software (version 3.69; Felsenstein, 1993). The principal component analysis (PCA) was conducted by GCTA software (Yang et al., 2011).

### Variant Filtering and Sliding Windows Analysis

To ensure the accuracy of the SNP index, the bi-allelic variants for individual M_2_ population with reads ≥ six-fold coverage depth in both bulks were retained. Before further analysis, variants including SNPs and short indels were filtered in three steps. In Step 1, we hypothesized that the EMS-induced mutations should be generated randomly, and therefore should be population specific. We removed common variants detected in ≥2 populations as background variation and mutations specific to only one M_2_ population were retained. In Step 2, the SNP index of variants was calculated for both bulks of a population. The SNP index was calculated for a locus as the ratio of non-reference reads to the total reads mapped to a variant locus. In any population, the variant loci with SNP index > 0.7 in both bulks were removed as they could probably be population-specific background mutations unlinked to the locus for the mutant phenotype. In Step 3, variants with SNP index < 0.3 in both DNA bulks of a population were also removed as the low proportion of the non-reference reads increased the probability of spurious variants resulting from sequencing or alignment errors. The SNPs, in repulsion phase, segregating with the wild-type bulk with SNP index = 0 in mutant bulks were eliminated.

The remaining SNPs were subjected to sliding window analysis. The difference between SNP indices [delta SNP index (DSI)] of a locus was calculated as the difference between SNP indices of two bulks for a locus in an M_2_ population. The fitted curve of SNP index or DSI (including absolute and non-absolute values) was obtained by averaging the values from a moving window of 10, 20, or 40 consecutive SNPs and shifting the window one SNP at a time. The optimal number of consecutive SNPs to fit the curve was selected according to the density of SNP retained in each population. The *x*-axis value of each window was set at a midpoint between the first and the last SNP. After identifying the genomic region harboring the candidate causal variant, we considered all variants (including SNPs and indels) in that region as the candidate causal mutations.

To simulate the effect of chimera, we introduced *n*% (*n* = 0, 10, 20, …, or 90) progenies without mutagenic variants from the causal cell into wild-type bulk of the Mut04 M_2_ population. This simulation led to declining of the SNP index of wild-type bulk to 1–*n*% of the original value. The DSI and DSI distribution were plotted for each simulation level. DSIs under different simulation levels were also adjusted by zero-centering. In this process, each DSI was subtracted from the mean of DSI along the genome, and then absolute value of DSI (ADSI) was calculated from the adjusted DSI. Finally, the plots of adjusted DSI and DSI distribution were drawn for each simulation level.

### Multiple-Sequence Alignment and Phylogenetic Analysis

The homologous genes of *RHD3* were searched from Phytozome and *Saccharomyces* Genome Database for the following genomes: *G. max*,^1^^,^^2^ *Arabidopsis thaliana*, *Medicago truncatula*, *Trifolium pretense*, *Phaseolus vulgaris*, and *Saccharomyces cerevisiae S288C*. The phylogenetic tree was constructed by using MEGA 7.0 software. The neighbor-joining method was applied to construct the trees with the bootstrap method with the bootstrap value of 1,000 (Kumar et al., 2016).

### Validation of Mutation in *Gmrhd3*

Genomic DNA was isolated from the leaf tissue of *Gmrhd3* and wild-type IGA 1008 by using the cetyl trimethylammonium bromide (CTAB) method (Murray and Thompson, 1980). PCR was used to validate the mutation in the *GmRHD3*. PCR primers were designed by using the genomic sequence of *Glyma.08G193200*. The sense primer was 5'-TTCTTCCTCATTAGTAGCCAGTATAG-3' and the antisense primer was 5'-AAACCATAGCGTCATTACCGTG-3'.

### Scanning Electron Microscopy and Image Analyses

The leaf and petiole at the V4 stage were used for scanning electron microscopy (SEM) analysis. The images were captured by using a JSM-IT500 microscope with an acceleration voltage of 10 kV. The length of trichome was calculated by ImageJ software (Schneider et al., 2012).

### Code Availability and Implementation

All software codes of the M2-seq approach were implemented in perl, and the code and its detailed usage are available in GitHub.^3^

The corresponding M2-seq pipeline has also been developed as a free online analysis platform. Users can carry out the analysis by uploading SNPs information of your materials in Variant Call Format (VCF). The website of M2-seq is www.omicshare.com/M2-seq.

## Results

### Principles of M2-Seq

**Figure 1:**
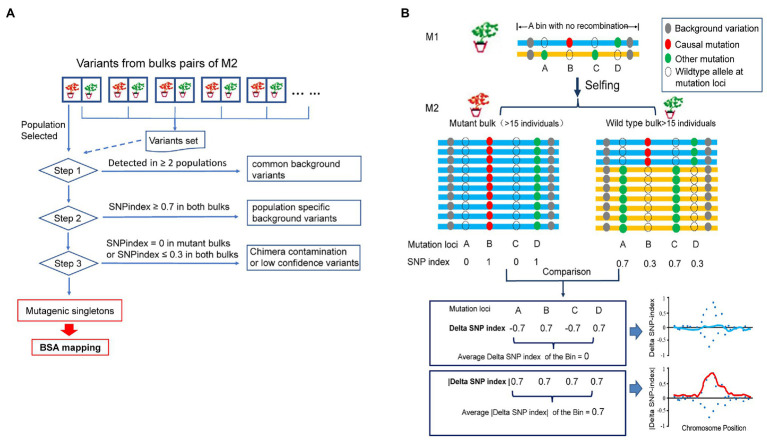
A simplified scheme of M2-seq. **(A)** Overview of the process of variant filtering. **(B)** Mapping a genomic region harboring a causal variant. The curve of absolute value of delta single-nucleotide polymorphism (SNP) index (red curve) is used to identify the region of a causal mutation (for details, see text).

The principles of M2-seq are illustrated in Figure 1. EMS-induced individual M_1_ soybean mutants were self-pollinated to generate M_2_ populations. M_2_ populations potentially harboring recessive causal mutations using at least 15 mutant progenies were selected to clone the target gene. The occurrence of chimera, plant harboring more than one genotype after the mutagen treatment, is a common phenomenon among the mutants. By studying the segregating ratios of the wild-type to mutant individuals in M_2_ generation, one can predict the number of initial mutagenic cells involved in generating the seeds of M_1_ plants. If there was no gametic selection or embryo lethality, the expected ratio of wild type to mutant in M_2_ population should be equal to (4 k - 1): 1, with k being the number of initial mutagenic cells (Spencer-Lopes et al., 2018). For example, if the seeds of M_1_ plant were from single initial cell, then a ratio of wild type to mutant, 3:1, expected; otherwise, 7:1 for progenies descending from two initial cells resulting in chimerism (Koornneeff et al., 1982). For each M_2_ population, two DNA bulks, wild-type and mutant bulks, originating from 15 individuals with wild-type and mutant phenotypes, respectively, were generated. WGS with high reads depth (>30 fold) was conducted for each bulk. The M2-seq is comprised of two key processes: (1) removal of undesirable variations and (2) mapping of the genomic regions that harbor the causal mutations.

In order to purge most of the undesirable variations representing the genetic polymorphisms between the progenitor of the mutants and reference genome sequence or sequencing/alignment error, we designed a variant-filtering process for the data from multiple M_2_ populations as follows (Figure 1A). In Step 1, population-specific variants are kept, and background variations identified in two or more M_2_ populations are purged. In Step 2, the SNPs and indels with SNP index > 0.7 in both DNA bulks of an M_2_ population are removed as they are individual M_2_ population-specific background variations. In Step 3, SNPs and indels with SNP index < 0.3 in both DNA bulks of an M_2_ population were removed as they are spurious variations derived from a sequencing or an alignment error. Any variants with SNP index = 0 in the mutant bulks are also eliminated in this step as they could be potential variants obtained from chimeric mutations, originating from a separate mutagenic cell. After filtering all undesirable variations in the above steps, the population-specific singletons were retained for identifying the candidate mutation(s) governing the mutant phenotype.

The bioinformatics approach taken here for mapping the genomic regions harboring the causal mutations is different from that used in the Mutmap method. The approach is, however, similar to that applied for BSA of the pseudo-test cross population (Xue et al., 2017). In the Mutmap method, most of the mutagen-induced mutations in the mutant parent are fixed (homozygous) through several generations of selfing before the mutant is hybridized to the wild-type parent. The direction of segregation distortion for DSI close to the causal mutation is consistent, and therefore the fitted curve of DSI assisted in mapping the region harboring the causal mutation. EMS-induced mutations are generated independently in the M_1_ genome and can lead to potential inconsistent linkage phase between the causal mutant allele and the nearby mutagen-induced mutant alleles. In M2-seq as shown in Figure 1B, mutagen-induced mutations are heterozygous in M_1_, and the mutation alleles can be located on either of the two homologous chromosomes. Therefore, the selection of causal mutant allele at locus B in the mutant bulk led to the diverse SNP index for the nearby linked mutant loci. For example, the mutant allele at locus D, located next to locus B in coupling phase linkage, will be selected along with the causal mutant allele at locus B during bulking; and therefore the SNP index of locus D could be the same as that of the locus B (Figure 1B). The mutant alleles at loci A and C located on the other homologous chromosome in repulsion phase linkage; and thus the selection of causal mutant allele in locus B led to an enrichment of the wild-type alleles at the two mutant loci in the mutant bulk. Therefore, their SNP index for loci A and C would be zero or close to zero in the mutant bulk. In the wild-type bulk of genotypes with the wild-type phenotype, wild-type allele’s dominance over the mutant allele was observed at B locus and D locus while the mutant allele was dominant at A locus and C locus. In our above example, although the DSIs of causal mutation locus B is positive, mutations in linked loci can be positive (as in locus D), or can be negative if the mutation is in repulsion to the causal mutation (e.g., loci A and C). For a majority of the BSA methods, the mean value of DSI of consecutive variants within a bin (genomic region of a given length) are calculated and fitted to a curve to reduce the effect of random fluctuation for DSI of single variants, and the peak of the curve is identified as the candidate region harboring the causal mutation. Generally, the expected mean values of DSI should be convergent to zero even for the region linked to causal mutation as two opposite DSIs exist within this region as stated above. In M2-seq, we express the DSI in absolute values, and then the ADSI is used to plot the chart and to identify the candidate region.

### Application of M2-Seq in 10 Soybean M_2_ Populations

From screening progenies of 2,200 M_1_ plants, 10 independent M_2_ populations carrying visible morphological mutants were selected for this study (Table 1). The size of the M_2_ populations ranges from 85 to 267 (Table 1). The wild-type: mutant ratios range from 2.81:1 to 10.61:1. Of these, seven showed 3:1 segregating ratio (*p* > 0.05) for mutations in a single initial cell model. Two populations, viz. Mut06 and Mut10, showed distorted segregating ratios that fitted the 7:1 ratio (*p* > 0.05) for the generation of the progenitor M_1_ plants from two cells and one population Mut09 fitted the ratio of 11:1 (*p* > 0.05) suggesting that the progenitor M_1_ was evolved from three cells.

**Table 1:** Phenotype ratio of progeny in 10 M2 populations.

Cross ID	Mutant	Wild type	Total	Observed ratio	Expected ratio			Mutant phenotype
					3:1^a^	7:1^b^	11:1^c^	
Mut01	16	69	85	4.31:1	0.188^d^	0.078	4.67E-04	Short trichome, dwarf
Mut02	29	96	125	3.31:1	0.642	2.98E-04	1.81E-09	Wrinkled leaves
Mut03	37	109	146	2.95:1	0.924	2.70E-06	1.04E-13	Etiolation for leaves, dwarf
Mut04	26	73	99	2.81:1	0.772	3.46E-05	1.09E-10	Wrinkled leaves
Mut05	22	81	103	3.68:1	0.393	0.007	1.73E-06	Slight etiolation for leaves
Mut06	22	102	124	4.64:1	0.062	0.078	1.50E-04	Etiolated and wrinkled leaves, dwarf
Mut07	61	178	239	2.92:1	0.852	1.15E-09	6.91E-22	Etiolation for young leaves
Mut08	26	78	104	3.00:1	1	1.16E-04	7.77E-10	Etiolation for leaves, dwarf
Mut09	23	244	267	10.61:1	6.28E-10	5.50E-02	0.868	Narrow leaflets
Mut10	23	139	162	6.04:1	0.001	0.51	0.01	Leaf petiole angle increasing, etiolation and high temperature sensitive

a *p*-value for one-cell model. Segregation of wild-type to mutant alleles in M_2_ populations was tested for the 3:1 ratio with the hypothesis that the respective seeds of M_1_ were developed from single initial cell; *p* values in this table were calculated for the chi-square values.

b *p*-value for two-cell model. Segregation of wild-type to mutant alleles in M_2_ populations was tested for the 7:1 ratio with the hypothesis that the respective seeds of M_1_ were developed from two initial cells.

c *p*-value for three-cell model. Segregation of wild-type to mutant alleles in M_2_ populations was tested for the 11:1 ratio with the hypothesis that the respective seeds of M_1_ were developed from three initial cells.

d Yellow background color in tables indicates the best model for the corresponding population.

The leaves collected from either wild-type or mutant individuals were used to generate wild-type and mutant DNA bulks, respectively, for each of the 10 M_2_ populations (see Section “Materials and Methods” for details). Whole-genome resequencing was conducted for each DNA bulk with a read depth of >30-fold (Supplementary Table S1). After variant calling and quality control, we identified a total of 340,546 mutations including 294,181 SNPs and 46,365 short indels among the 10 pairs of bulks. A phylogenetic tree was constructed for the SNPs detected in all 20 bulks. The phylogenetic tree showed that the bulks from the same M_2_ population clustered tightly (Supplementary Figure S1). The reference genome Williams 82 cultivar formed a separate cluster in the tree. The outcome of the PCA also supported the results of the phylogenetic tree (Supplementary Figure S2).

**Figure 2:**
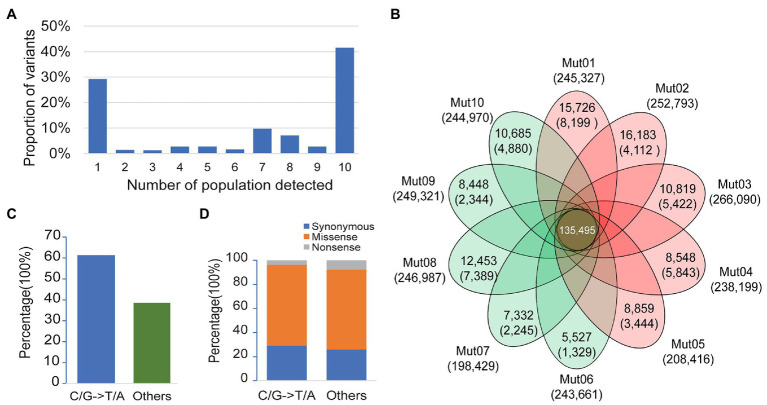
Patterns of genetic variation among 10 populations. **(A)** The allele frequency spectrum of 340,546 variants detected from 10 bulk pairs highlights that a high proportion of the genetic variants are population specific (present in only one population) or widespread (present in all 10 populations). **(B)** The Venn plot of the number of high-quality variants (HQ variants) detected in each population. The total number of HQ variants within each population is listed below the population name. The number of HQ variants shared by all populations is in the center. Numbers without the parenthesis in the non-overlapping portions of each oval indicate the number of HQ variants unique to each population, while the numbers in the parenthesis represent the number of ethyl methanesulfonate- (EMS-) induced variants in each population. **(C)** Proportion of canonical and noncanonical EMS-induced mutants in all populations. **(D)** Proportion of mutagenic variants in a coding region with different functional classes.

Among the 340,546 variants detected, 29.2% were singletons (variants seen only once in one of the 10 bulk pairs), 41.5% were common variants (variants seen in all the 10 bulk pairs), and the remaining 29.3% consisted of variants detected in 2–9 bulk pairs (Figure 2A). We applied a stringent depth filter (depth ≥ 6 in both bulks of a pair) to define high-quality variants (HQ variants) in each pair. The number of variants detected in each bulk pair was 239,419 ± 20,472 (Supplementary Table S2).

EMS-induced mutation rate is about 1–10 mutations/Mb (Schneeberger et al., 2009; Doitsidou et al., 2010; Zuryn et al., 2010; Abe et al., 2012; Xiao et al., 2019), and the genome size of soybean is about 1 Gb. Thus, the expected number of EMS-induced variants would be about 1,000–10,000 in each pair. The variants detected here in each population were about 20-fold more than the expected number of mutagenic variants. Taken together, we speculate that a majority of the variants detected in each pair were non-mutagenic, although the wild-type parental lines IGA 1008 were derived from Williams 82, which is the cultivar used to generate the reference genome.

The non-mutagenic variants were removed with three steps as a method mentioned above. The number of variants retained or removed in each step is shown in Figure 2B and Supplementary Table S2. After Step 1 of filtering the common background variants, a total of 5,572–16,183 singletons were retained in each pair (Figure 2B). After Steps 2 and 3 for the removal of population-specific background and spurious or chimera contamination variants, the numbers of variants retained decreased to 4,521 ± 2,258 (Figure 2B). The density of retained variants was about 1–8/Mb (the size of the reference was regarded as 1 Gb), which was consistent with the expected range of the EMS mutagenesis rate described above. Finally, a total of 51,986 variants were retained from 10 pairs of bulk as mutagenic variants, of which 51,409 were single-nucleotide variants (SNVs). Only 61.4% of the mutagenic SNVs were canonical EMS-induced transition-type (C/G>T/A) while the remaining 38.6% non-C/G>T/A SNVs were classified to others (Figure 2C). This result was consistent with the composition of EMS-induced mutations reported previously (Sarin et al., 2010). In the coding genes, both canonical and noncanonical type of EMS-induced SNVs contained a high proportion of missense and nonsense mutations (Figure 2D). Thus, the non-C/G>T/A mutagenic mutations should not be ignored in the genetic analysis of the mutant.

**Figure 3:**
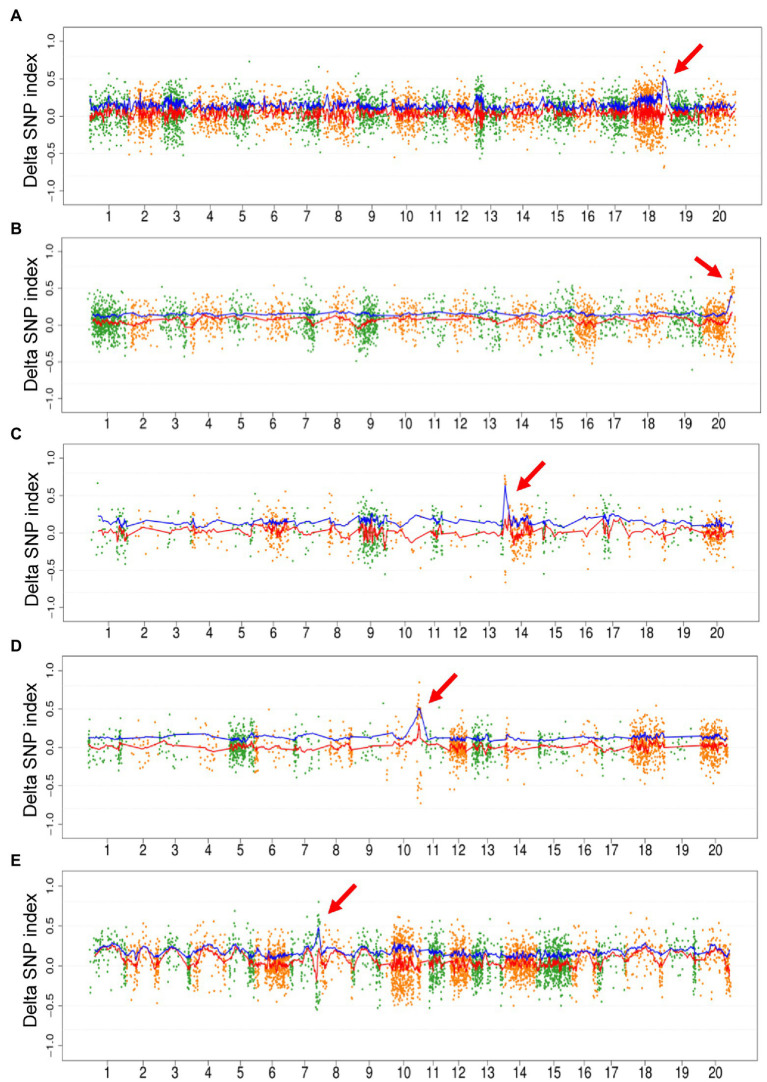
Plot of M2-seq mapping of five M_2_ populations. The M_2_ populations included Mut03 **(A)**, Mut05 **(B)**, Mut06 **(C)**, Mut07 **(D)**, and Mut10 **(E)**. Each point represents a single-nucleotide variant (SNV), the red line is the fitted curve of delta SNP index (DSI), and the blue line is the fitted curve of absolute value of DSI (ADSI). The red arrows indicate the candidate regions detected by the curve of ADSI.

**Figure 4:**
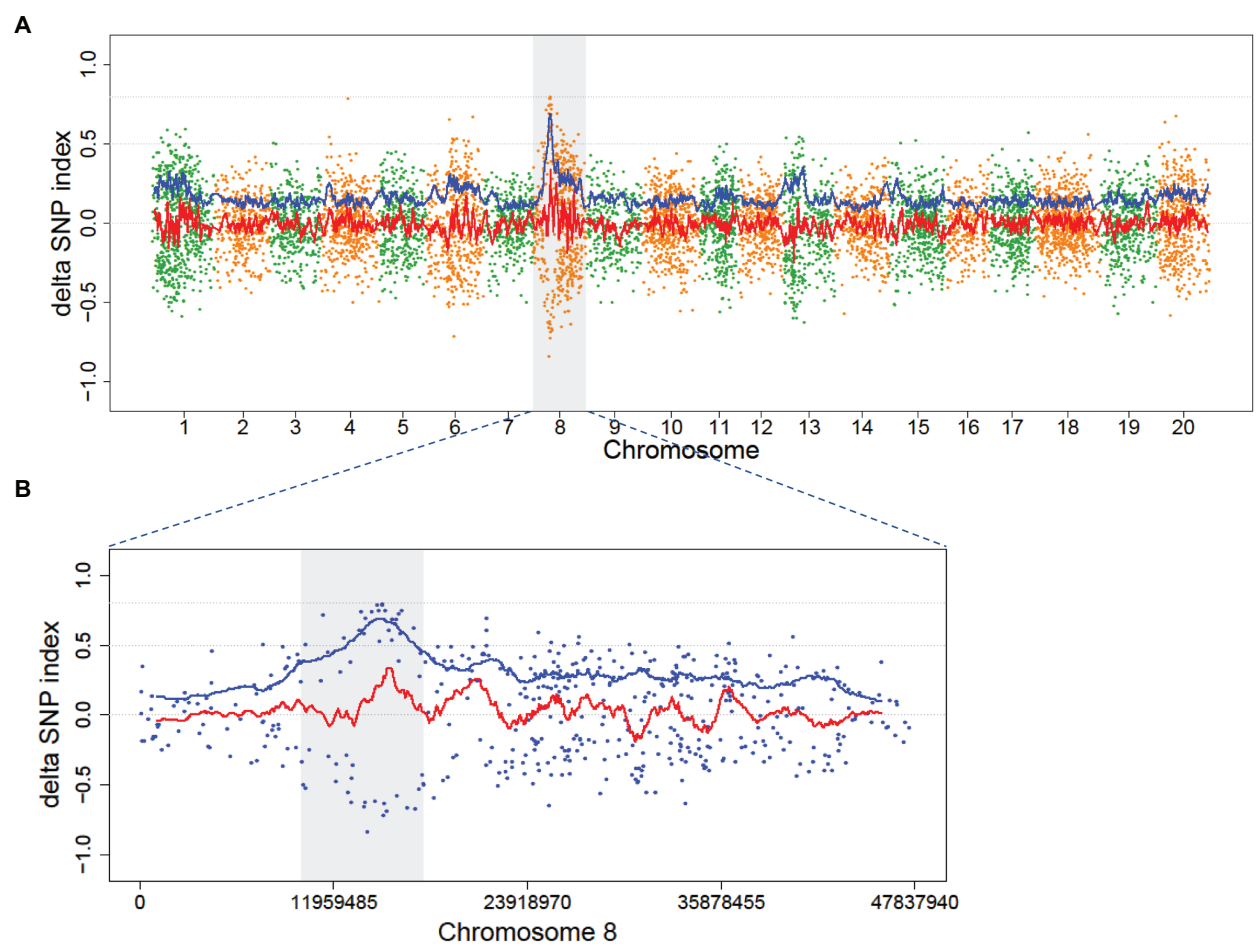
Distribution of ADSI in the M_2_ of Mut01 population at the whole-genome level and causal region in Chromosome 8. Each point represents a SNVs, the red line is the fitted curve of DSI, and the blue line is the fitted curve of ADSI. **(A)** Whole-genome plot, Chromosome 8 is highlighted with gray color. **(B)** Partial enlarged view of Chromosome 8, the candidate region (10–18 Mb) is highlighted with gray color, the peak of blue line indicates the candidate region with a causal variant.

As the proof-of-principle experiment mentioned above, we only used mutagenic SNPs to map the region of causal mutation. The fitted curve of DSI and ADSI was plotted for all the 10 populations. No distinct peak was detected on the curve of DSI in 5/10 populations, including Mut03, Mut05, Mut06, Mut07, and Mut10. On the contrary, all ADIS curves showed unique peaks in these five populations (Figure 3). In the remaining five populations, although the DSI curves displayed detectable peaks harboring causal mutations, the corresponding signals in the ADSI curves were clearer than DSI (Supplementary Figure S3, e.g., for Mut01 see Figure 4). These results confirmed that the repulsion phase linkages of the causal mutations with mutations in adjacent regions seriously weaken the signals in DSI curve. In the ADSI curves, the use of absolute values of the DSI assists mapping of the region more effectively. In all 10 populations of this study, the peak regions were detected on the ADSI curves (Supplementary Table S3) and laid the foundation for gene identification.

### Identification of the Candidate Causal Mutation in Population Mut01

**Figure 5:**
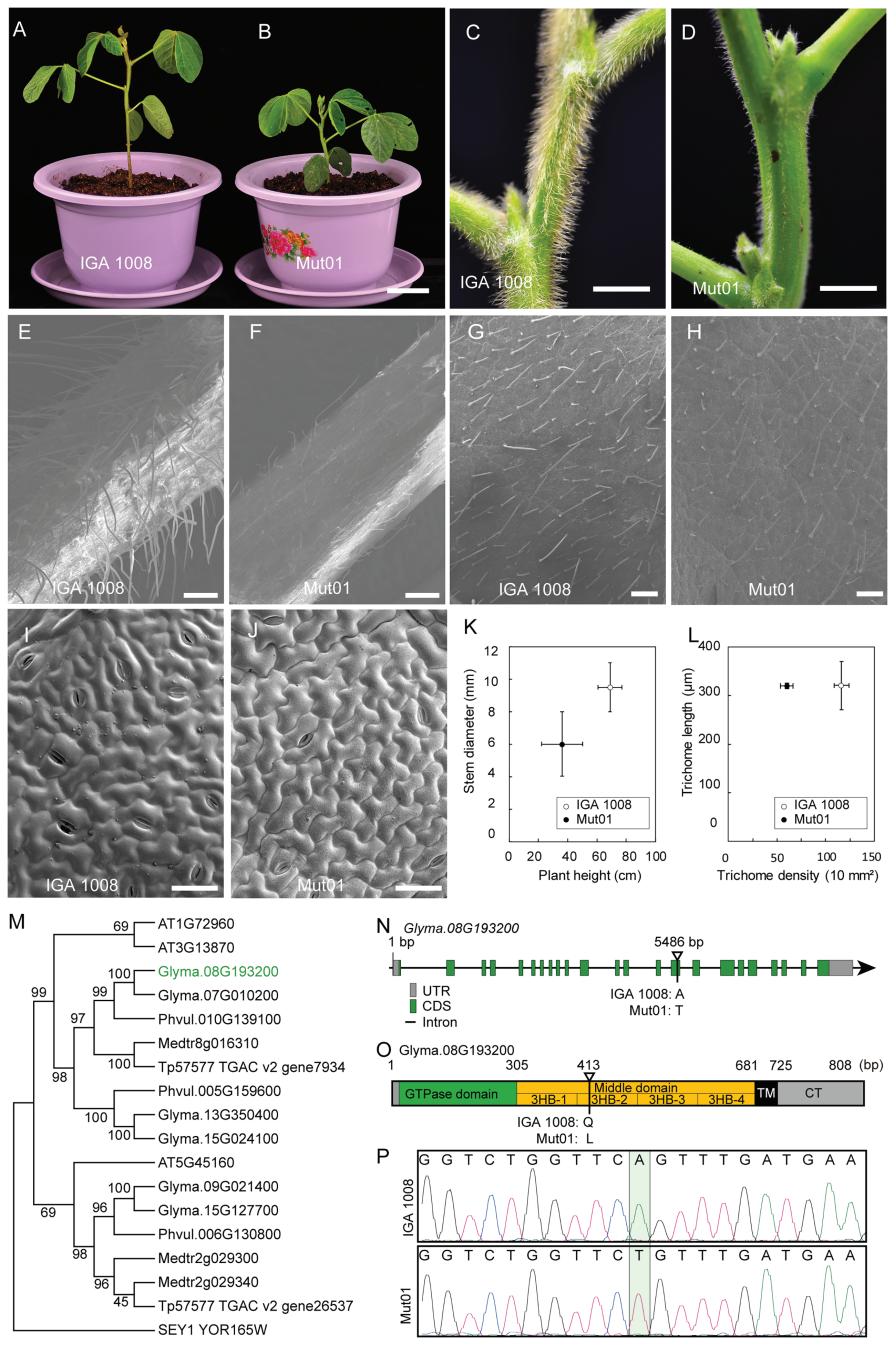
Characterization of phenotype of mutant from Mut01 and causal mutation in gene *Glyma.08G193200*. **(A,B)** Mutant and wild-type IGA 1008 phenotype of whole plants at V4 stage. Scale bar, 5 cm. **(C,D)** Mutant and wild-type phenotype of stem. Scale bar, 5 mm. **(E,F)** Mutant and wild-type phenotype of petiole. Scale bar, 500 μm. **(G,H)** Mutant and wild-type phenotype of leaf trichome. Scale bar, 500 μm. **(I,J)** Mutant and wild-type phenotype of the leaf pavement cell. Scale bar, 50 μm. **(K)** Plant height and stem diameter of mutant and wild type. Values are mean ± SD (*n* = 6 plants). **(L)** The trichome length and density of the mutant and wild-type leaf. Values are mean ± SD; 150 trichomes were used to calculate the mean values of trichome length of mutant and wild type. **(M)** The phylogenetic tree of RHD3 was obtained from *Arabidopsis*, *Glycine max*, *Medicago truncatula*, *Phaseolus vulgaris*, *Trifolium pretense*, and *Saccharomyces cerevisiae*. *Glyma.08G193200* was marked with green. **(N)** Schematic illustration of the genomic locus of *GmRHD3*. Exons and introns are shown in boxes and lines, respectively. Mutation site for the *Gmrhd3* was indicated. **(O)** The overall structure of full-length *GmRHD3*. The number on the top indicates the corresponding amino acid positions. The triangle represents the mutation site of *Gmrhd3*. **(P)** DNA sequencing peak chromatograms of genomic DNA of *Gmrhd3* and wild type close to the mutation site. The mutation site was marked with pale green rectangle.

The target mutant phenotype studied in the population Mut01 was dwarf plants and glabrous stem, petiole, and leaf as compared to the wild-type IGA 1008 (Figures 5A–J). The plant height of the mutant, 36.2 ± 5.7 cm, was significantly lower than that of the wild-type plant height, 69.0 ± 8.2 cm (*p* < 0.01). The stem diameter of the mutant was 6.01 ± 1.50 mm, which was smaller than that of the wild type, 9.52 ± 1.52 mm (*p* < 0.01; Figure 5K). The trichome length of the leaf in the mutant and wild type was 320 and 321 μm, respectively, with no significant difference (Figure 5L). However, the trichome density of leaf in the mutant, 60/10 mm^2^, was lower than that of wild type, 116/10 mm^2^ (*p* < 0.01; Figures 5G,H,L). The petiole and stem trichomes in the mutant were less abundant and shorter than that of the wild-type trichomes (Figures 5C–F). However, the size of leaf pavement cell of the mutant did not differ significantly from that of wild type (Figures 5I,J).

The causal variant in Mut01 was mapped to the 10–18 Mb region on Chromosome 8 (Figure 4). The region harbored 16 EMS-induced mutations in protein-coding gene region (16 SNVs and 0 indels; Supplementary Table S4). Of these, 13 mutations were C/G > T/A transition-type. Of the 16 mutations, 10 had positive DSI while the remaining 6 had minus DSI. This phenomenon explained the reason for higher signal in the ADSI curve than the DSI in this region. We examined four SNPs with SNP index = 1 in the mutant bulk and ADSI > 0.5. The four SNVs were nonsynonymous mutations localized to four protein-coding genes.

Among these four genes, *Glyma.08G193200* is most likely to be the candidate gene controlling the trichome density (Figure 5). *Glyma.08G193200* is a homolog of the Arabidopsis *AT3G13870* and *AT1G72960* genes (Figure 5M) sharing 76.5 and 77.0% identity with the two Arabidopsis genes. *AT3G13870* and *AT1G72960* belong to the *Arabidopsis root hair defectives 3* (*AtRHD3*) gene family consisted of three genes (Hu et al., 2003). Therefore, we propose that *Glyma.08G193200* is a homolog of *AtRHD3*. *RHD3* plays a major role in mediating the fusion of homotypic endoplasmic reticulum (ER; Zhang et al., 2013). The maintenance of ER integrity by GTP-dependent ER fusion genes might be crucial in cells with long protrusions (Hu et al., 2009, 2011). The loss of *AtRHD3* caused short and wavy root hair, a small rosette, and dwarf phenotype by reducing leaf size and stem length in *Arabidopsis* (Wang et al., 1997; Yuen et al., 2005; Zhang et al., 2013). The *AtRHD3* mutant carrying a non-synonymous point mutation in *AtRHD3* exhibited a very severe growth phenotype than the null mutant because the mutant *AtRHD3* protein exerts a dominant-negative effect (Zhang et al., 2013). The consistency between the phenotype of the population Mut01 and *AtRHD3* mutants indicated that the mutation in *Glyma.08G193200* was the causal mutation for the observed phenotype in Mut01. The causal mutation (A–T transversion) located at the 5,486 bp of *Glyma.08G193200* (Figure 5N). This result was verified by sequencing of the PCR product amplified from *Glyma.08G193200* (Figure 5P). The A–T transversion in *Glyma.08G193200* led to the substitution of glutamine with leucine. *GmRHD3* consisted of a cytosolic N-terminal GTPase domain (GD), three-helix bundles (3HB) enriched middle domain, two TM segments, and a cytosolic C-terminal tail. The mutation occurred in the second 3HB (3HB-2) of the middle domain (Figure 5O), which is critical for the efficient ER membrane fusion (Sun and Zheng, 2018). According to the above information, we inferred that the mutation in *Glyma. 08G193200* is most likely to be candidate causal mutations for the Mut01 mutant phenotype.

### Identification of the Causal Mutation in the Mut07 Population

We investigated the mutations within the candidate region in the Mut07 population. The mutant phenotype investigated in the Mut07 population is characterized by yellow green first true leaves during early developmental stages (Supplementary Figure S4). The total pigment contents including chlorophyll and carotenoid in leaves of the mutant were less than that of the wild type. The content of thiamine in unifoliate leaves of the 8-day-old mutant seedlings was only 74.6% of that in wild type (Feng et al., 2019). The causal variant of Mut07 was mapped to a 7 Mb region on Chromosome 10 (Supplementary Figure S5). The region harbored 13 EMS-induced mutations in protein-coding gene region (12 SNVs and 1 indel; Supplementary Table S5). Of these, 10 mutations were C/G > T/A transition-type that were the canonical EMS-induced SNV type. Of the 13 mutations, seven had positive DSI while the remaining 6 had minus DSI. Among the mutations, one mutation with positive DSI was selected as the candidate causal variant because it is the only mutation with SNP index = 1 and ADSI > 0.5 (SNP index = 1 in mutant bulk, SNP index = 0.39 in wild-type bulk). This mutation was a single-nucleotide (T) deletion in the protein-coding *Glyma.10G251500* gene at 47,970,082 bp of soybean Chromosome 10. This deletion mutation occurred at 292 nucleotide position in *Glyma.10G251500* CDS, which resulted in a truncated protein because of a premature stop codon at the 357 nucleotides position (Supplementary Figure S5). To verify the mapping result and gene function of *Glyma.10G251500* gathered through M2-seq of Mut07, we isolated one M_2_ mutant plant from the Mut07 M_2_ population and hybridized the isolated mutant plant to the Hedou12 cultivar to generate an F_2_ segregating population. The same candidate region was mapped and the same candidate causal variant in *Glyma.10G251500* was detected within a candidate region in this F_2_ population. Furthermore, loss-of-function T1 heterozygosis transgenic lines were generated by inducing mutations in the *Glyma.10G251500* gene using a CRISPR/Cas9 system. The CRISPR/Cas9-induced mutations in *Glyma.10G251500* in two independent mutants caused the development of the Mut07-specific mutant phenotype (Feng et al., 2019). The results mentioned above suggested that the mutation in *Glyma.10G251500* is the causal mutation leading to the Mut07 mutant phenotype.

### Simulation of Additional Mutagenic Variant Effect in Wild-Type Bulk of Population

The wild-type bulk is contaminated by additional mutagenic variants from initial noncausal mutagenic cells while the mutant bulk only contains the mutagenic variants from the initial causal mutagenic cell. The additional mutagenic variants from noncausal mutagenic cells need to be removed to avoid their interference in the subsequent mapping process while the mutagenic variants from the causal cell need to be retained as markers for mapping. The characteristic of the former variants is SNP index = 0 in the mutant bulk and >0 in the wild-type bulk while the characteristic of the mutagenic variants from the causal cell is SNP index > 0 in both bulks. Thus, the two types of mutagenic variants can be separated in Step 3 of the current variant-filtering process. As a result, only mutagenic variants from the causal cell will be retained for the next step of BSA mapping.

**Figure 6:**
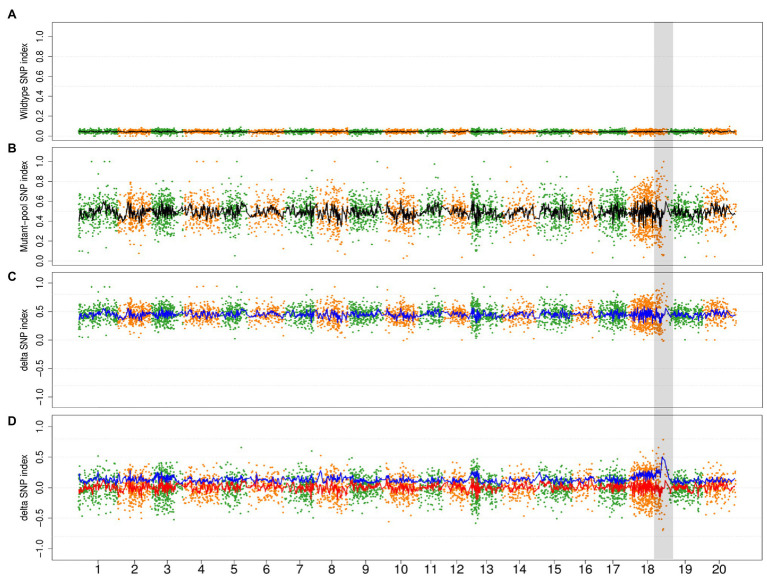
The simulation result of 90% progenies in wild-type bulk of population Mut03 from noncausal mutagenic cells. **(A,B)** The distribution of SNP index in wild type and mutant bulks in whole-genome level. Each point represents a SNV, and the black line is the fitted curve of SNP index, the candidate region is highlighted with gray color. **(C)** The distribution of DSI and ADSI. Each point represents a SNV, the red line is the fitted curve of DSI, and the blue line is the fitted curve of ADSI, while two curves overlap completely in this plot. **(D)** The distribution of adjusted DSI (zero-centering) and ADSI. Each point represents a SNV, the red line is the fitted curve of DSI, and the blue line is the fitted curve of ADSI.

In order to evaluate the impact of the mutagenic variant effect, we conducted a simulation wherein 0–90% of the progenies in wild-type bulk of the Mut03 population were derived from the noncausal mutagenic cells. Owing to the decreased genetic ratio of causal mutagenic cells by noncausal mutagenic cell, the SNP index of the retained variants (variants from causal mutagenic cell) in wild bulks declined (Figure 6A) and the average value of DSI increased to >0 (Figure 6C). In the event that all the DSIs were >0, ADSI would be no longer effective (only negative value could be adjusted by absolute value). Therefore, we adjusted the value of ADSI by zero-centering (subtracting mean value of ADSIs along the genome). After zero-centering of DSI, the causal region could be correctly mapped by ADSI at different chimeric levels (Figure 6D; Supplementary Figure S6).

## Discussion

### M2-seq, a Rapid Method of Gene Cloning in Plants

In this study, we have shown that one can identify candidate genes just in M_2_ generation through analyses of WGS of two bulked DNA samples: (1) mutant bulk carrying recessive mutations in homozygous condition and (2) wild-type bulk carrying the mutations in heterozygous condition. The SNP index (DSI) calculated by comparing the sequences of individual bulks with the reference genome sequence is used to identify the genomic regions carrying the causal mutations in target genes. We have shown that the application of absolute DSI (ADSI) eliminates the background variation resulting from repulsion phase linkages or chimeric origin of linked mutagen-induced mutations in plots of DSI values; and as a result, the detection of genomic regions carrying the target genes becomes feasible. In our study, we observed that the use of ADSI allowed us to detect the target genomic regions for all 10 causal mutations as opposed to only five, when the DSI values were used to map the target genomic regions (Figure 3; Supplementary Figure S3). The M2-seq method has been successfully applied in identifying soybean genes (Figure 5; Supplementary Figure S5; Feng et al., 2019). As opposed to many selfing generation required in previously reported WGS-BSA methods, such as Mutmap and Mutmap+, the M2-seq method can identify genes just in M_2_ generation. This means by applying the M2-seq method one can save the time required for map-based cloning of plant genes. This method will particularly impact gene cloning in plants that have longer generation time. The method is suitable for cloning those genes, mutagen-induced recessive alleles of which produce mutant phenotype. Embryo lethal mutations and dominant mutations may unlikely be considered for this approach.

For WGS-based BSA methods, increasing the number of progenies in each bulk is beneficial to improve the mapping accuracy. In this study, we adopted the strategy of collecting only 15 progenies in each bulk, which mainly considered the characteristics of soybean M_2_ population from EMS mutagenesis. The number of progenies with mutant phenotype in M_2_ population was limited due to some reasons, such as finite number of seed harvested from single M_1_ plant, germination rate, and the existence of chimeras (Table 1). Therefore, we set the standard protocol of M2-seq as collecting 15 progenies in each bulk. The candidate interval still could be determined with the 15-progeny bulks in the studied M_2_ populations (Supplementary Table S3). In the case of a limited number of progenies in each bulk, the mapping interval of our M2-seq study was generally larger (the size of mapping interval was 2–10 Mb in most populations, and even reached 23 Mb in the Mut08 population). The reasons for detecting the candidate causal mutations using M2-seq are more likely due to the sufficient sequencing coverage of both bulks, the feature of EMS mutagenesis, and the powerful filtering algorithm. The average sequencing coverage depth of both wild type and mutants is 36.81, from 29.46 to 42.35 (Supplementary Table S1). The mutation rate of EMS mutagenesis was about 1–8 mutations/Mb for our materials and only a small amount of mutations was located in the coding gene region (Figure 1 and Supplementary Table S2). In the M2-seq method, the range of candidate causal mutations could be strictly screened out with two filtering criteria: (1) focusing only on mutations that could change the amino acid sequence of proteins; and (2) screening based on mutation frequency: SNP index = 1 in the mutant bulk and ADSI > 0.5. We detected 1–4 candidate causal mutations in 9 of our 10 M_2_ populations (Supplementary Table S3). No causal mutation was found in the Mut03 population, which may be due to incorrect gene mapping or false negative in variation calling of sequencing data. In the practical application, we recommend to collect more progenies to build bulks, which are conducive to narrowing the positioning interval.

### Importance of Removing Background Variants for Mutmap-Like Method

In the WGS-based BSA method, the segregated SNPs are used as markers to map the region harboring causal variants. In QTL-seq, the genetic difference between the parents is usually large. Genetic differences between unrelated soybean cultivars are about 1.894% (Lam et al., 2010). The WGS of parental lines is invaluable to identify the SNPs (Lu et al., 2014; Das et al., 2015). In Mutmap and Mutmap+, mutants are hybridized to the wild-type progenitor plant of the generated mutants to develop segregating population for BSA using the induced mutations. In addition to mutagen-induced mutations, a considerable number of background variants can be inherited from the progenitor of the mutant population, even if the genome sequence of the same cultivar is used as the reference genome.

In this study, IGA 1008 derived from cultivar Williams 82 was used to generate the mutant population. A comparison of the genome sequences of 10 pairs of bulked DNA samples originating from 10 M_1_ plants with the reference Williams 82 genome sequence revealed that a large amount genetic difference still exists between IGA 1008 and the reference Williams 82 genome sequence. We detected, on an average, 239,419 ± 20,472 variants among the 10 M_2_ populations. The average number variants induced by the EMS treatment was only 4,521 ± 2,258 (Supplementary Table S2). We infer that the number of background variants will reach 100 times, even 1,000 times more than mutagenic mutations when the wild-type progenitor is a distantly related variety compared to the reference genome. As most background mutations are fixed and not segregated in mapping populations, their DSI should be close to zero. If the background variants are not removed before BSA mapping, the identification of the genomic regions causal mutations will be affected by background variants leading to the failure in the detection of causal mutations of target genes. Therefore, the removal of background variants is critical for Mutmap-like methods.

The background mutations could be eliminated by WGS of the wild-type progenitors of the mapping populations. However, the wild-type progenitors may not be preserved after mutagenesis. Furthermore, the WGS of progenitors is not cost efficient. The current study demonstrated that the common background mutations could be effectively eliminated by comparing sequences of multiple bulks developed from different M_1_ plants generated from the same progenitor line (Figure 1A). We investigated if the Step 1 was essential for the removal of background mutations prior to mapping target genes. Without Step 1, the average number of remaining SNPs or mutations was 93,077 ± 4,720 among the 10 M_2_ populations (Supplementary Table S6), which was approximately 20-fold more than the number of variants retained if Step 1 was implemented. Theoretically, setting the threshold in Step 2 as SNP index ≥ 0.7 in both bulks should remove the common and population-specific background mutations simultaneously. However, we observed an unneglected level of false negative for removing the background mutations if only implementing Step 2; and therefore Step 1 is essential and cannot be replaced by Step 2. Thus, to identify background mutations, a comparison of multiple M_2_ populations is required. Having multiple independent M_2_ populations for the same target gene can provide multiple M_2_ populations for removing the background mutations and identifying the target gene with high confidence without conducting transformation. Thus, this method is expected to be very powerful in cloning genes based on mutant phenotypes governed by mutagen-induced recessive alleles.

Since NGS is widely applied, a large amount of WGS data is accumulated, which will be valuable to construct the variant database for the mutant study of different species. Exome Aggregation Consortium (ExAC) database is one of the largest human variant database containing >60,000 samples (Lek et al., 2016). The ExAC database is used as a reference to easily identify the *de novo* or rare variants for human genetic study. Construction of such a reference plant database will be useful in eliminating the background variations. In the plant mutant research, the mutant materials are usually generated from a limited number of representative cultivars; and thus a database contains even relatively fewer samples will be valuable to eliminate the background variants. A database containing 1,086 exon capture sequencing data of mutant maize lines generated from the B73 cultivar was constructed, which is an adequate reference for the cultivar B73-derived EMS mutant study (Lu et al., 2018). However, this database was constructed only based on exon capture sequencing, which only encompasses 82% of the protein-coding genes of maize. Insufficient coding gene coverage will increase the probability of false negatives for the causal mutation detection. Furthermore, for the WGS-based BSA study, background variants in the intergenic region should be eliminated while mutagenic variants in the intergenic region should be retained as markers for BSA mapping. However, intergenic variants cannot be detected by exon sequencing, and thus background variants in the intergenic region could not be eliminated by using exon sequencing-based database as a reference. With a continual decrease in the cost of NGS, the construction of a soybean mutant database based on WGS would be feasible.

A previous study focused on the canonical C/G > T/A EMS-induced mutation to narrow down the number of candidate mutations (Abe et al., 2012). However, in the current study, non-C/G > T/A mutagenic mutations constituted 38.6% of EMS-induced mutations, and the elimination of non-C/G > T/A mutation would increase the probability of false negative. Therefore, to explore the causal mutations among EMS-induced mutants, it will be necessary to analyze both canonical C/G > T/A and non-C/G > T/A mutations.

### Reason and Impact of Phenotypic Segregation Distortion

Among the 10 M_2_ populations we collected, the ratios of wild-type to mutant progenies range from 2.5–3.5:1 in five populations to >4:1 in the other five populations with the highest value of 10.61:1 for one M2 population. This phenomenon indicated that M_2_ generation tends to generate a higher number of wild-type progenies or lower number of mutant progenies than expected from the one initial cell model.

In addition to the potential effect of incomplete penetration or multi-causal mutations, we deduced the other two potential factors considering the characteristic of M_2_ generation (Supplementary Figure S7). First, a part of homozygous recessive mutation led to some degree of gametic selection or early development lethality, and thus the number of mutant progenies survive to the stage of phenotype evaluation was reduced. Second, as discussed earlier, mutants developed from two or more initial cells create chimerism leading to dilution of the desirable causal mutant allele in the seeds of M_1_ plant. This factor also increases the proportion of wild-type progenies in M_2_ generation.

The impact of these two factors in the outcomes of M2-seq is different. The first factor will only reduce the number of mutant progenies (with homozygous recessive mutation) harvested but not introduce new genetic composition. The SNP index values of the causal mutant loci will not be disrupted, and the mapping of the causal region is not affected. The impact of the second factor is rather complicated. Under the impact of second factor, the standard M2-seq strategy cannot detect the causal region. However, M2-seq with the zero-centering correction strategy can still identify the causal region, even if 90% of the offspring come from noncausal cells (Figure 6). This result indicated that the M2-seq is a robust procedure that can tolerate the impact of chimera from M1 generation.

### Repulsion Phase of Mutagenic Mutation

As described above, the mutagenic mutation can be generated in one of two homologous chromosomes randomly, which leads to repulsion phase linkages between adjacent mutagenic alleles. Therefore, conventional DSI method is not feasible to apply in such M_2_ generation. The proposed M2-seq method based on ADSI has been shown to be effective and robust to overcome such problems. The adjacent mutagenic alleles in repulsion phase linkage will be retained during selfing generations until mutagenic loci are fixed. The replacement of DSI with ADSI in Mutmap+ is expected to improve the detection of the genomic regions that carry causal mutations.

## Conclusion

In conclusion, we have developed an M2-seq method, which is an optimized and improved BSA method than the existing Mutmap and Mutmap+. In M2-seq, the phenotype observation and causal variant mapping could be performed in M_2_ generation, it only needs sufficient seeds produced from the M1 generation to observable phenotype in the M_2_ generation. With the continued decreases in the cost of WGS, M2-seq has a wide application in gene cloning, such as large-scale mutation mapping, especially for the species like trees that have long generation time.

## Data Availability Statement

The datasets presented in this study can be found in online repositories. The names of the repository/repositories and accession number(s) can be found at: https://www.ncbi.nlm.nih.gov/, SRP191330.

## Author Contributions

XF, MB, and SY designed the study. KT, YH, and GL performed the experiments. HZ, KT, and WL analyzed the data. XY established M2-seq online analysis platform. HZ wrote the manuscript. All authors contributed to the article and approved the submitted version.

### Conflict of Interest

WL was employed by company Guangzhou Gene Denovo Biotechnology Co. Ltd.

The remaining authors declare that the research was conducted in the absence of any commercial or financial relationships that could be construed as a potential conflict of interest.

## Footnotes

^1^https://phytozome.jgi.doe.gov/pz/portal.html

^2^https://www.yeastgenome.org

^3^https://github.com/FenglabBioinfo/DMAP_M2
