# Supplementary material for: A Robust and Rapid Candidate Gene Mapping Pipeline Based on M2 Populations
Source: Front Plant Sci. 2021 Jun 2;12:681816. doi: 10.3389/fpls.2021.681816 (PMC8207192; doi:10.3389/fpls.2021.681816)
Supplement: Supplementary file 1 [file Data_Sheet_1.doc]

**Supplementary FIGUREs and tables**


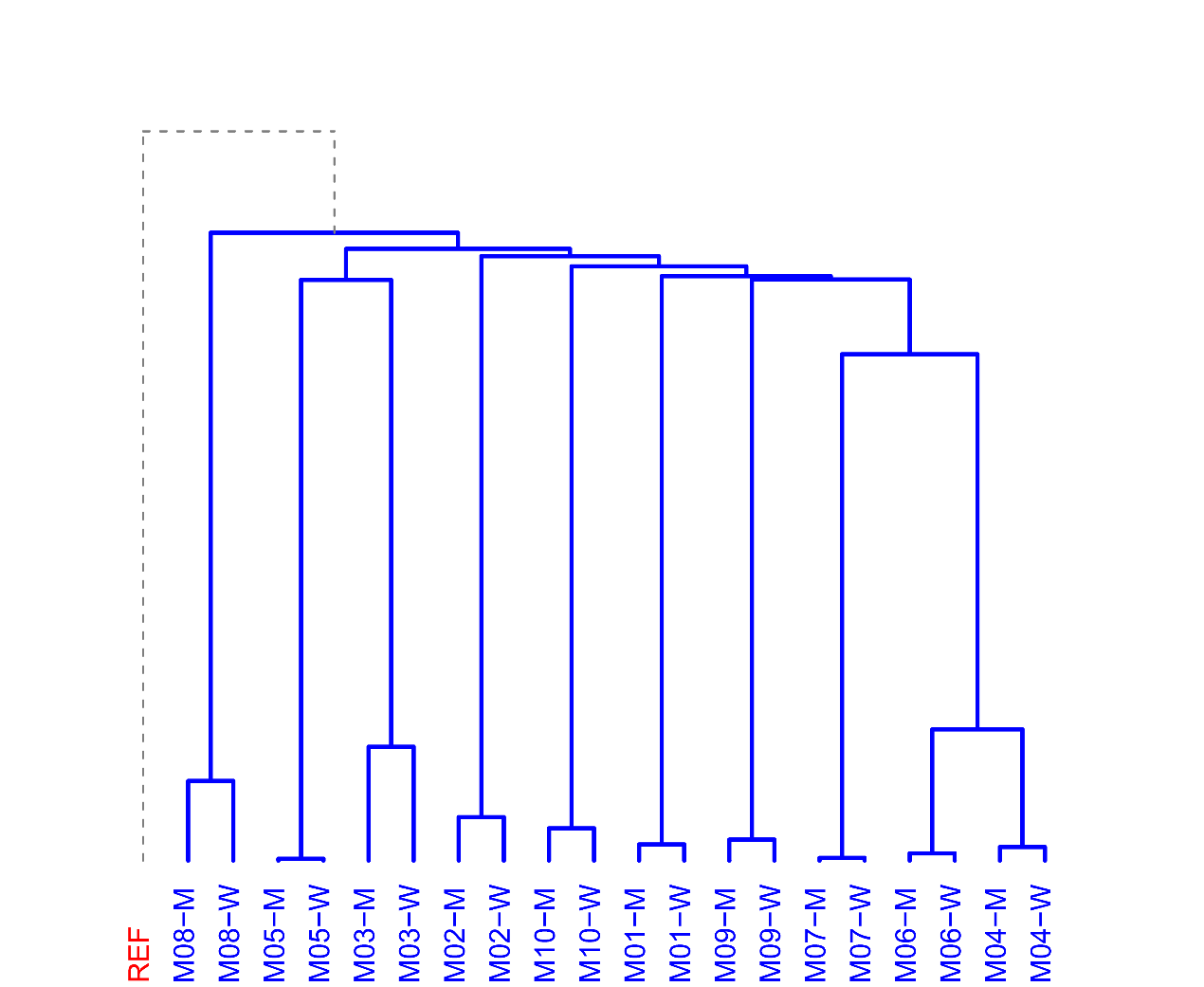


**FIGURE S1. A neighbor-joining tree constructed for 10 mutant and 10 wild-type bulks with the reference genome sequence using the SNP data.** REF, reference Williams 82 genome sequence; M08, M­2 population # 8; M, mutant bulk; W, wild-type bulk.


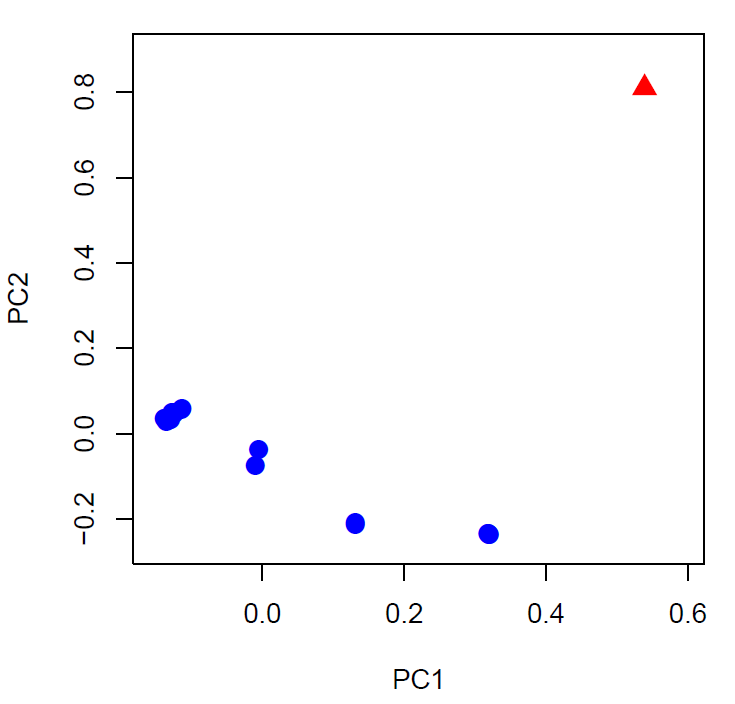


**FIGURE S2. Principal Component Analysis (PCA) for 20 bulks and reference genome using SNP data.** The red triangle represents the reference Williams 82 genome, and the blue circles represent bulk-pairs.


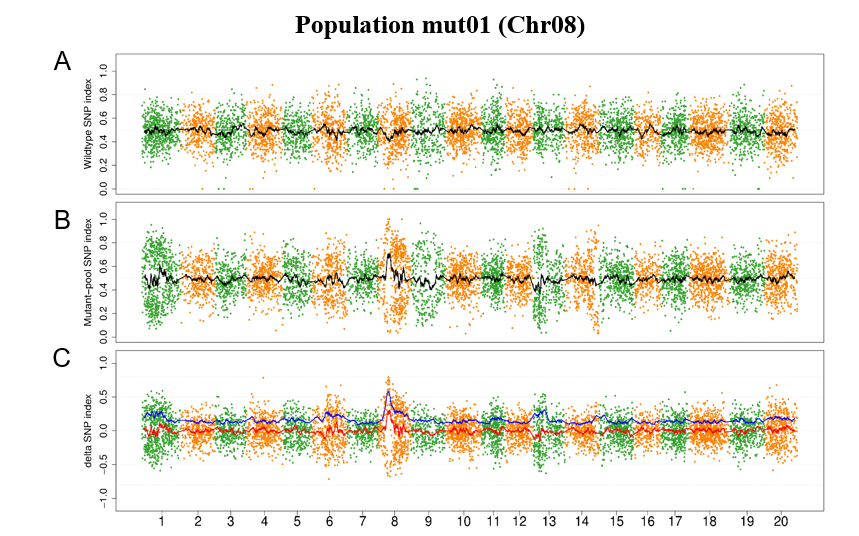


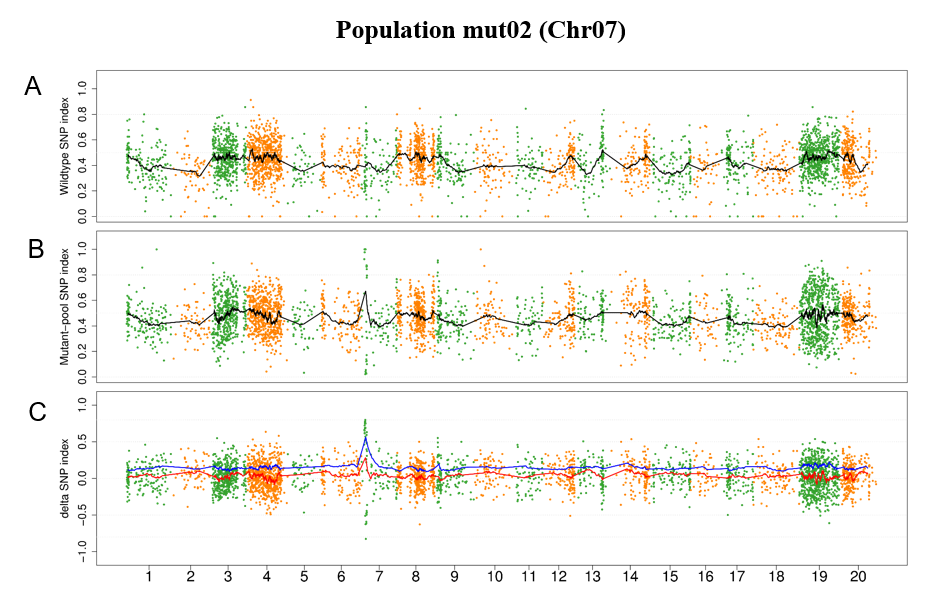


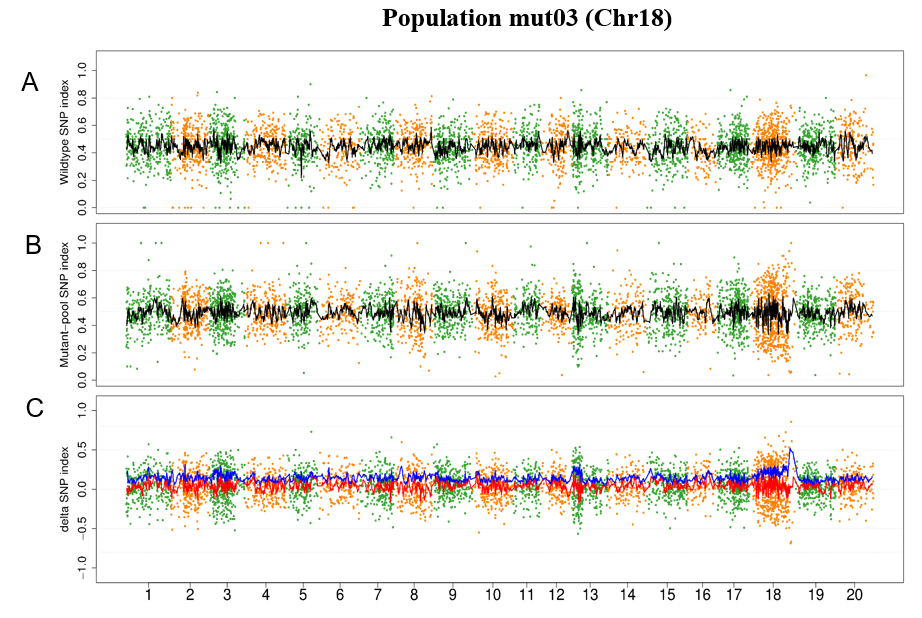


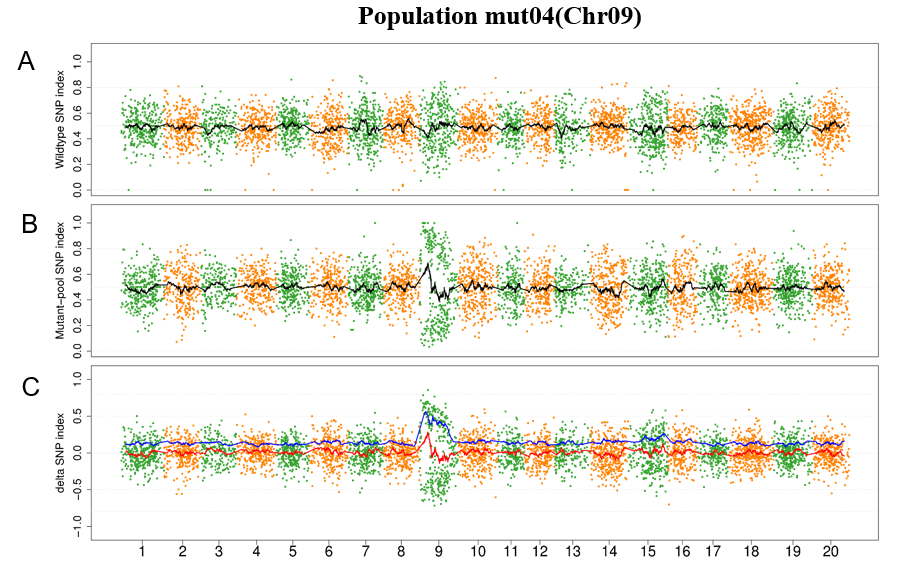


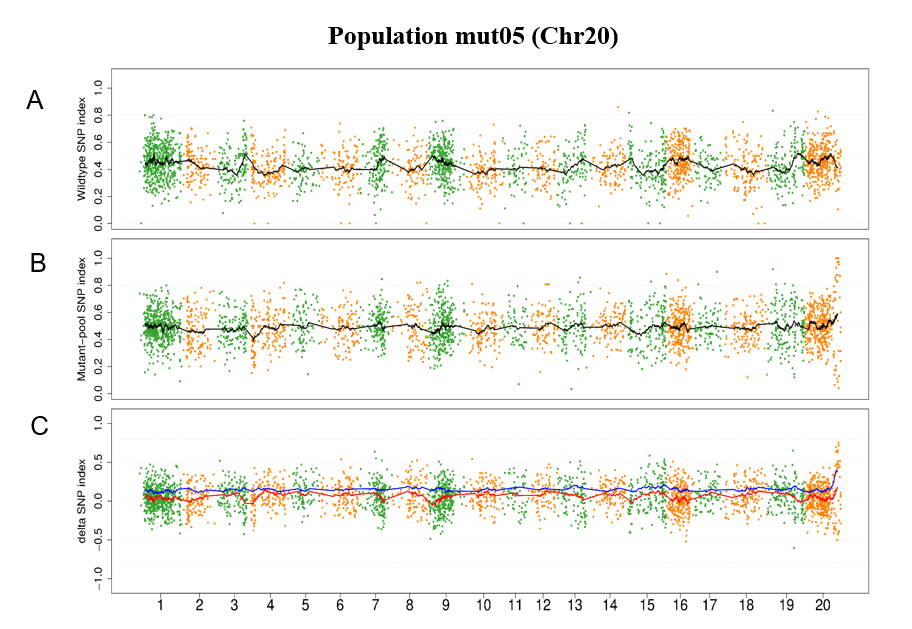


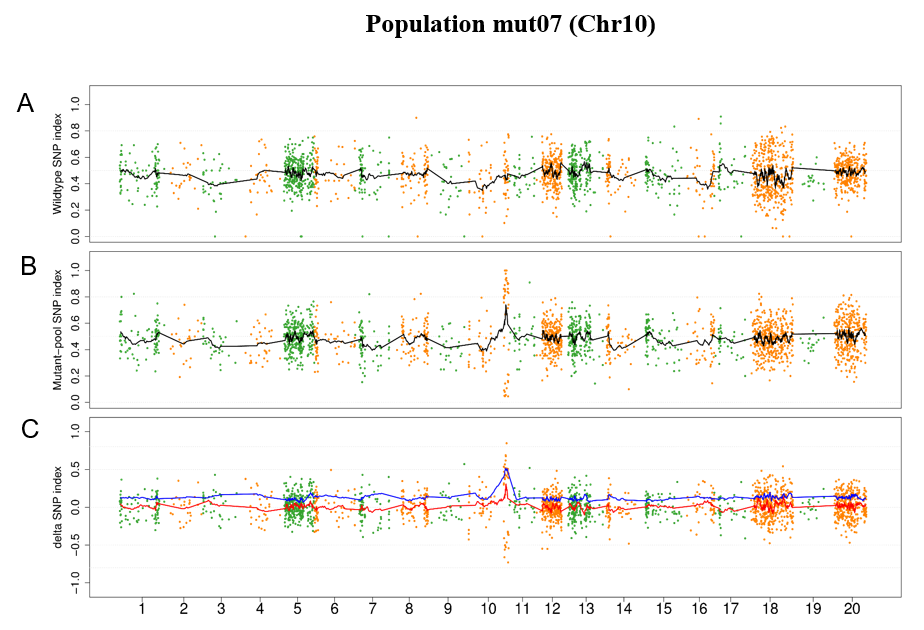


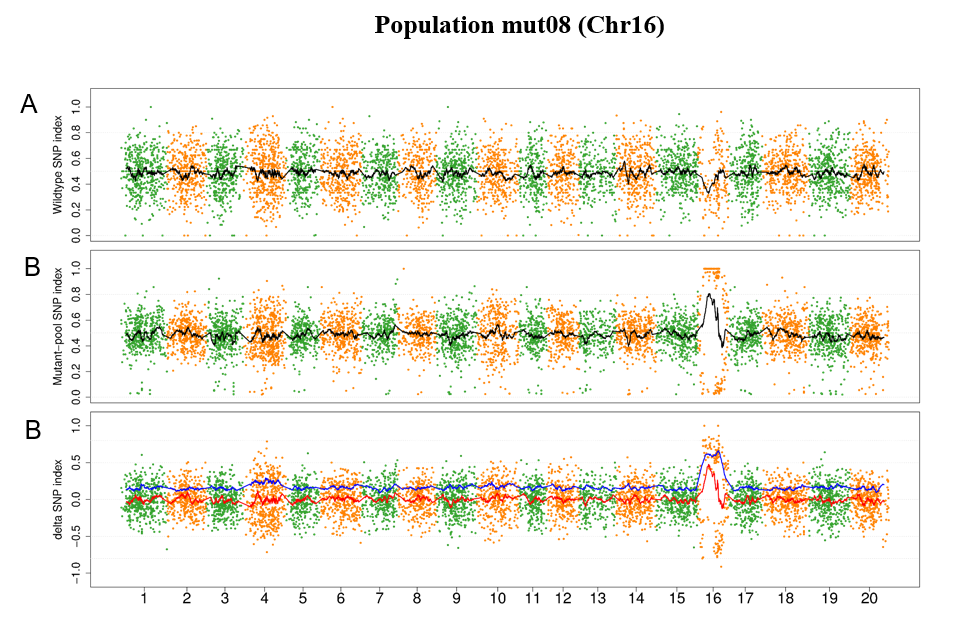


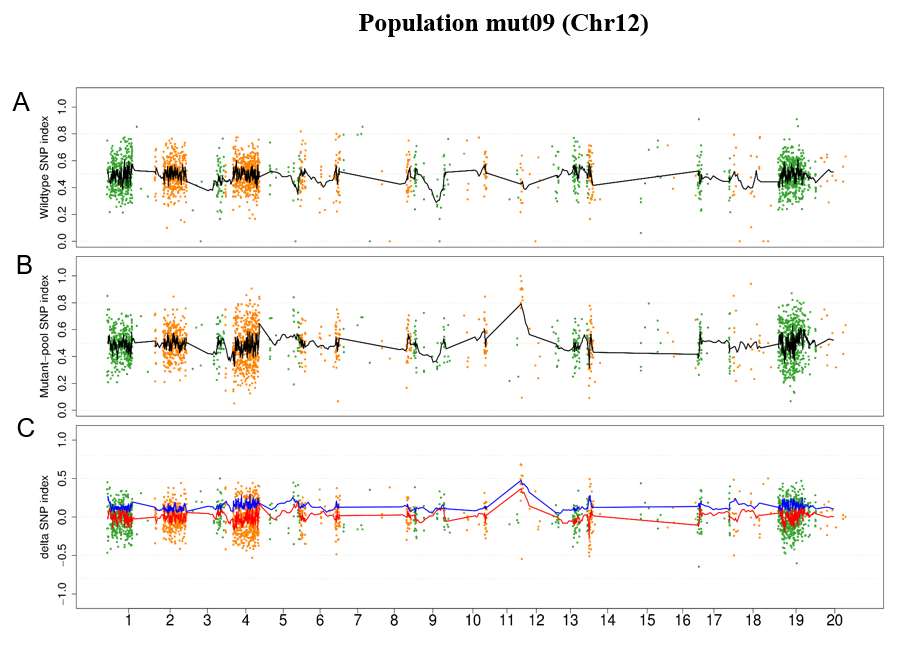


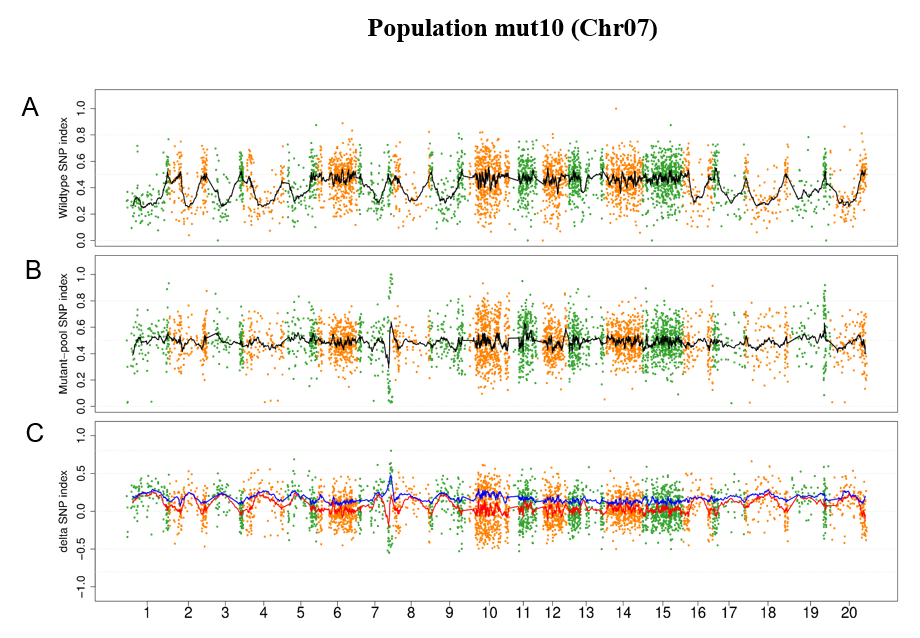


**FIGURE S3. The plot of M2-seq mapping for mutation populations.** The distribution of SNP index in (A) wild type and mutant (B) bulks in whole genome level. Each point represents a SNV, and the black line is the fitted curve of SNP index. (C) The distribution of SNP index of this population in whole genome level. Each point represents a SNV, the red line is the fitted curve of delta SNP index (DSI), and the blue line is the fitted curve of absolute value of DSI (ADSI). The chromosome ID with bracket in the headline of this FIGURE indicates in which chromosome the casual variant is located.


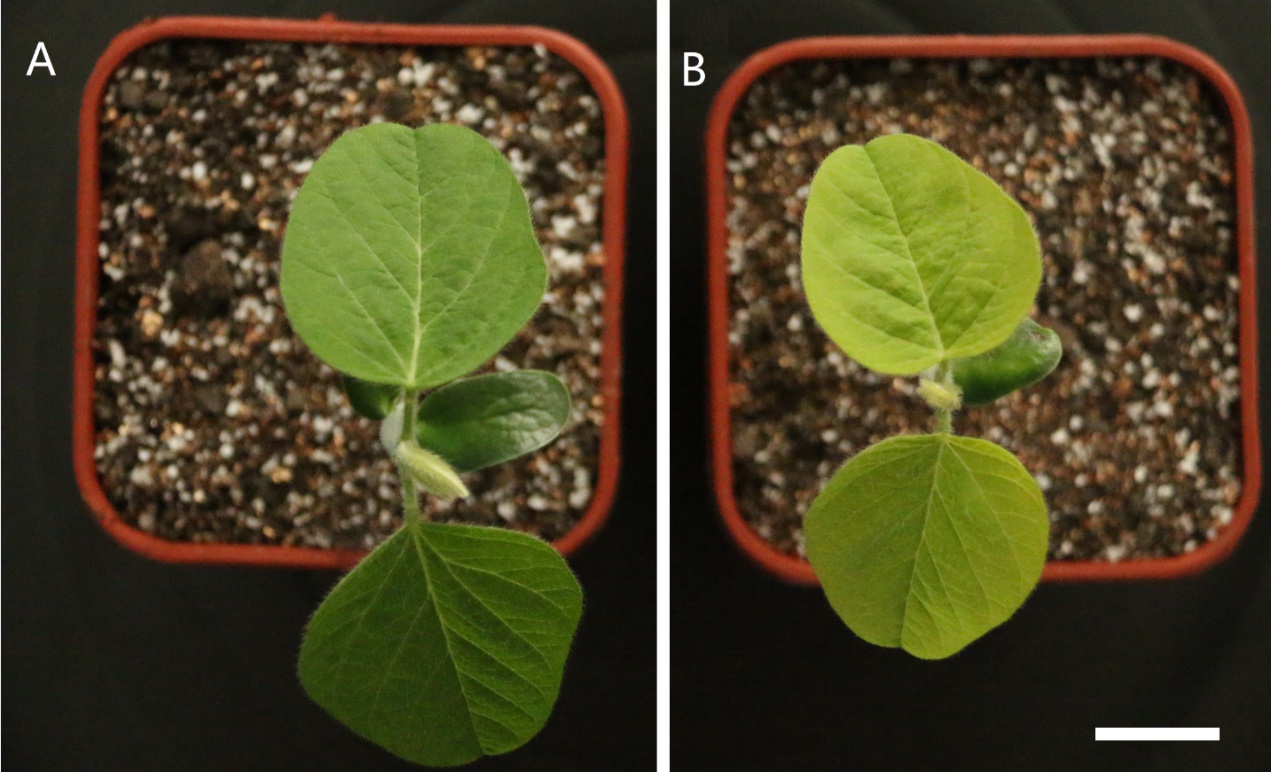


**FIGURE S4. Seedling phenotype of wild type (A) and Mut07 mutant (B).**


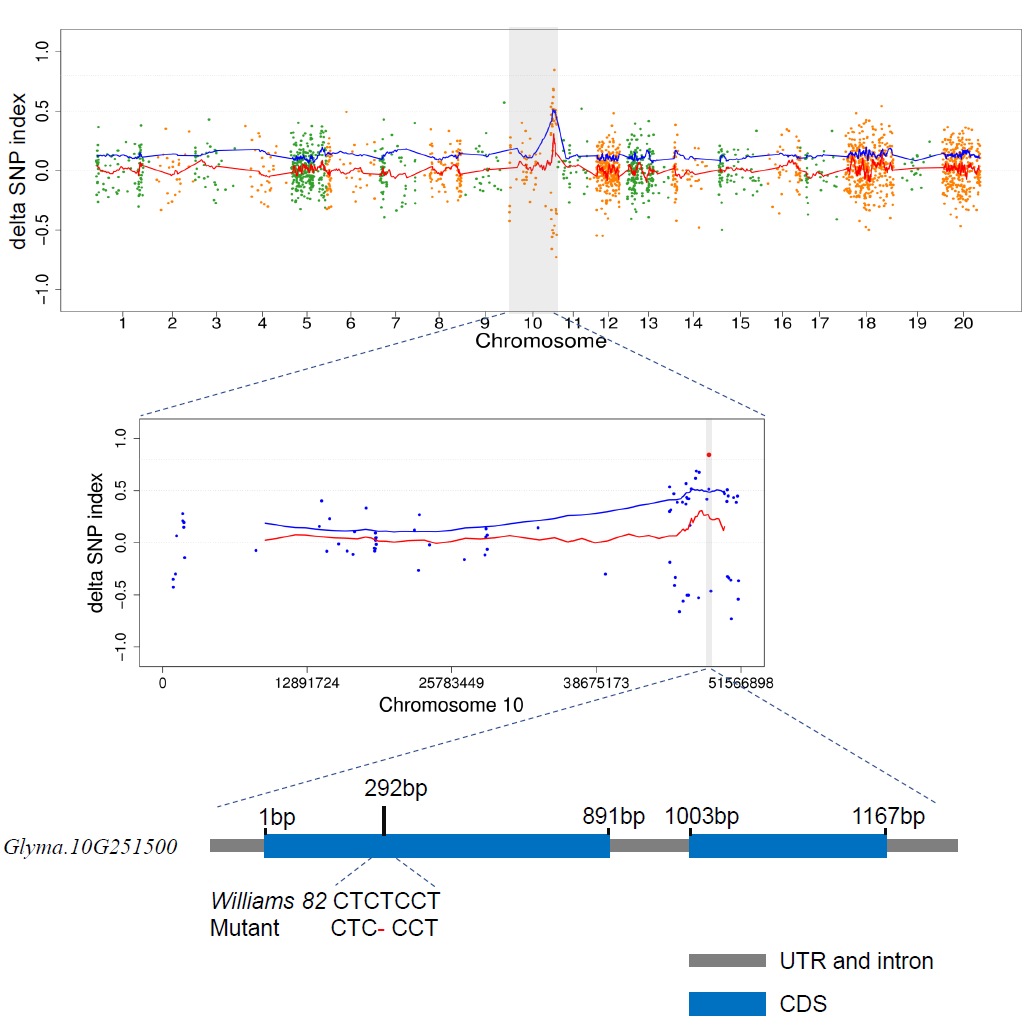


**FIGURE S5. The candidate region of Mut07 population and the causal mutation in *Clyma.10G251500*.**

Each point represents a (single nucleotide variants) SNV, the red line is the fitted curve of delta SNP index (DSI), and the blue line is the fitted curve of absolute value of DSI (ADSI). The peak in blue line indicates the causal variant region. The structure of candidate gene *Glyma.10G251500* is also shown. One thymidine nucleotide is deleted at 292 bp in the first exon of *Glyma.10G251500*


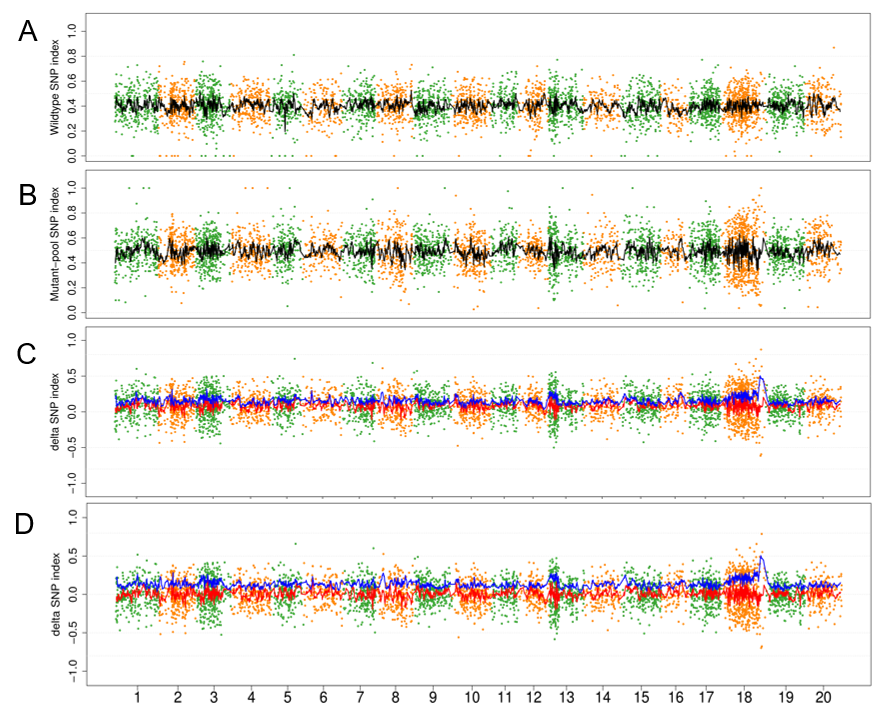


**FIGURE S6. The simulation results of various progenies in wild-type bulk of population Mut03 from non-causal mutagenic cells.** (1) 10% progenies derived from non-causal mutagenic cells. (A, B) The distribution of SNP index in wild type and mutant bulks in whole genome level. Each point represents a SNV, and the black line is the fitted curve of SNP index. (C) The distribution of delta SNP index (DSI) and absolute value of DSI (ADSI). Each point represents a SNV, the red line is the fitted curve of DSI, and the blue line is the fitted curve of ADSI. (D) The distribution of adjusted DSI (zero centering) and ADSI. Each point represents a SNV, the red line is the fitted curve of DSI, and the blue line is the fitted curve of ADSI.


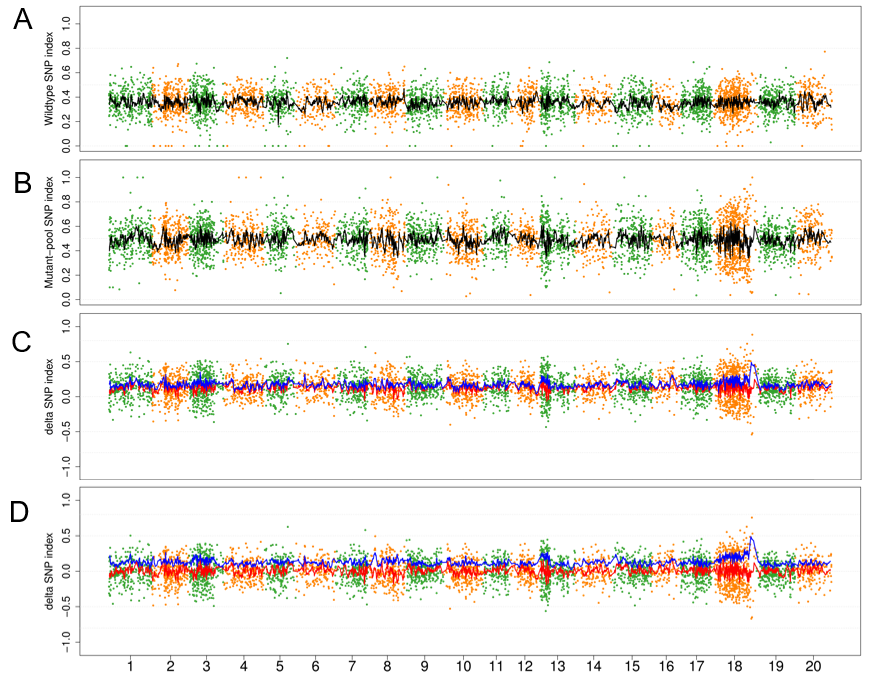


**FIGURE S6. The simulation results of various progenies in wild-type bulk of population Mut03 from non-causal mutagenic cells.** (2) 20% progenies derived from non-causal mutagenic cells**.** The rest as same as the simulation of 10% progenies.


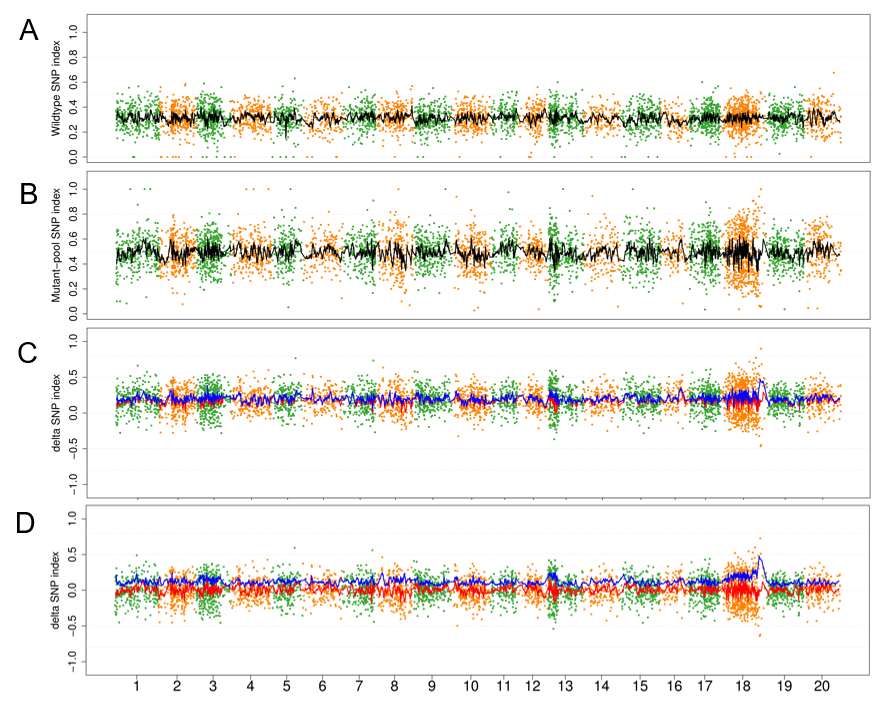


**FIGURE S6. The simulation results of various progenies in wild-type bulk of population Mut03 from non-causal mutagenic cells.** (3) 30% progenies derived from non-causal mutagenic cells**.** The rest as same as the simulation of 10% progenies.
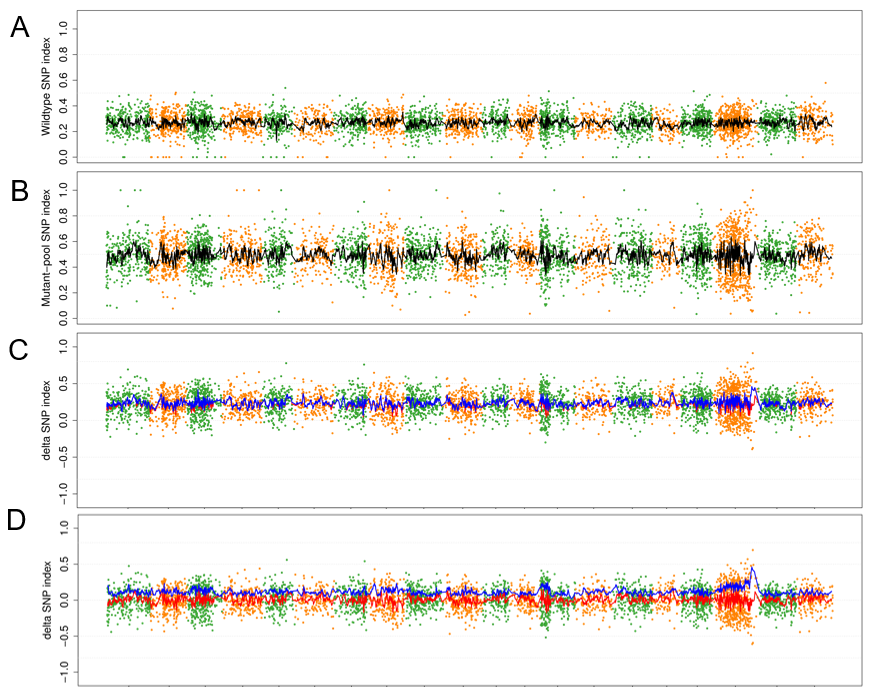


**FIGURE S6. The simulation results of various progenies in wild-type bulk of population Mut03 from non-causal mutagenic cells.** (4) 40% progenies derived from non-causal mutagenic cells**.** The rest as same as the simulation of 10% progenies.


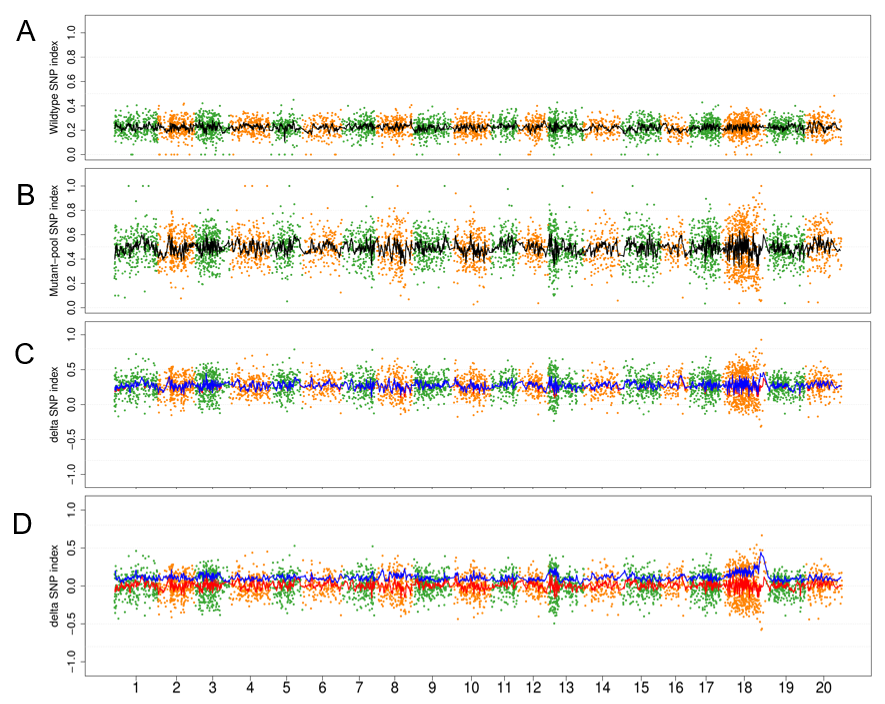


**FIGURE S6. The simulation results of various progenies in wild-type bulk of population Mut03 from non-causal mutagenic cells.** (5) 50% progenies derived from non-causal mutagenic cells**.** The rest as same as the simulation of 10% progenies.
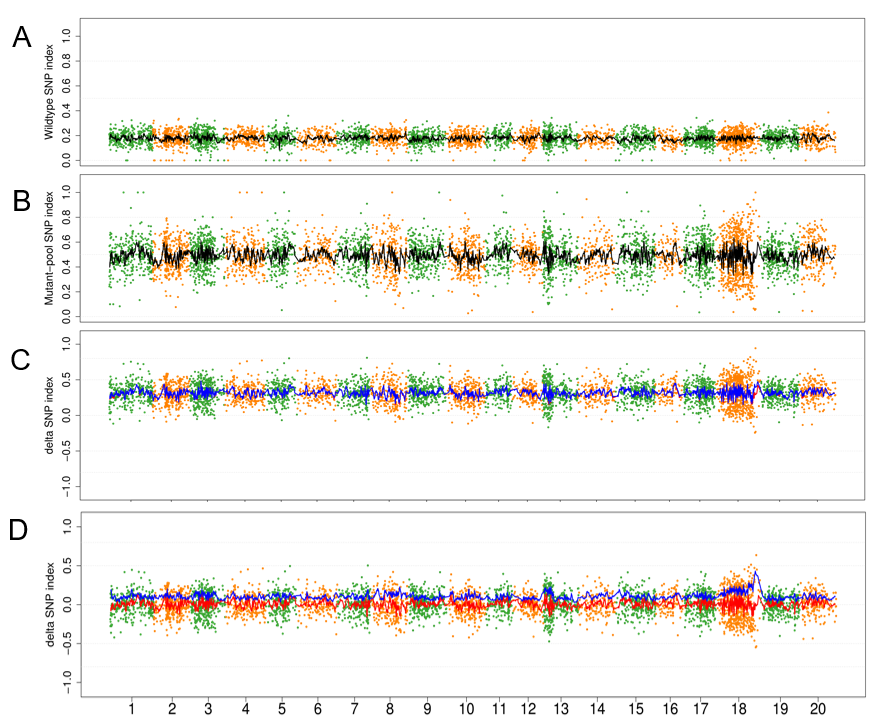


**FIGURE S6. The simulation results of various progenies in wild-type bulk of population Mut03 from non-causal mutagenic cells.** (6) 60% progenies derived from non-causal mutagenic cells**.** The rest as same as the simulation of 10% progenies.
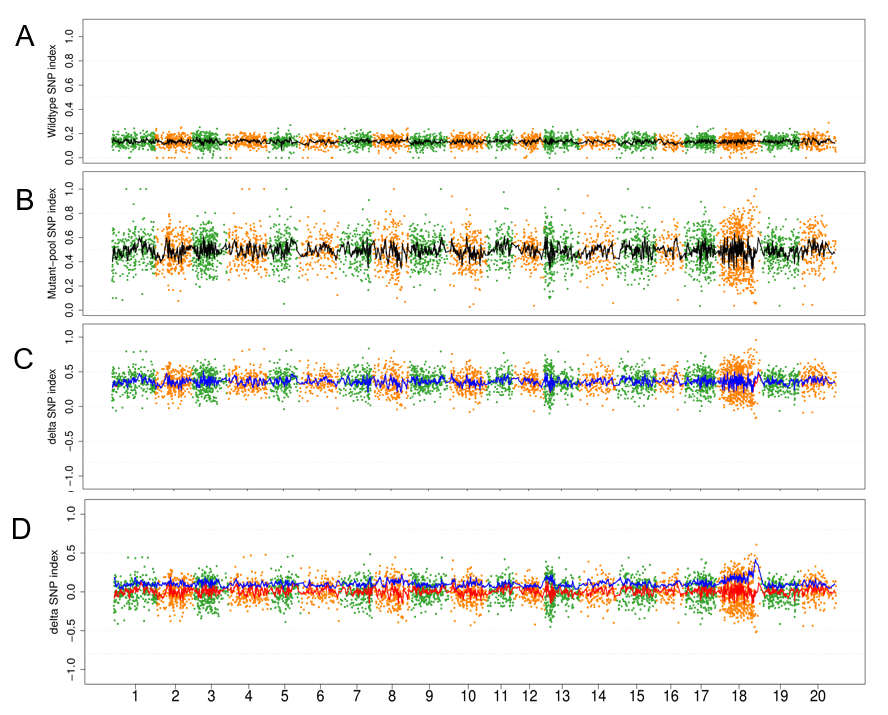


**FIGURE S6. The simulation results of various progenies in wild-type bulk of population Mut03 from non-causal mutagenic cells. (7)** 70% progenies derived from non-causal mutagenic cells**.** The rest as same as the simulation of 10% progenies.
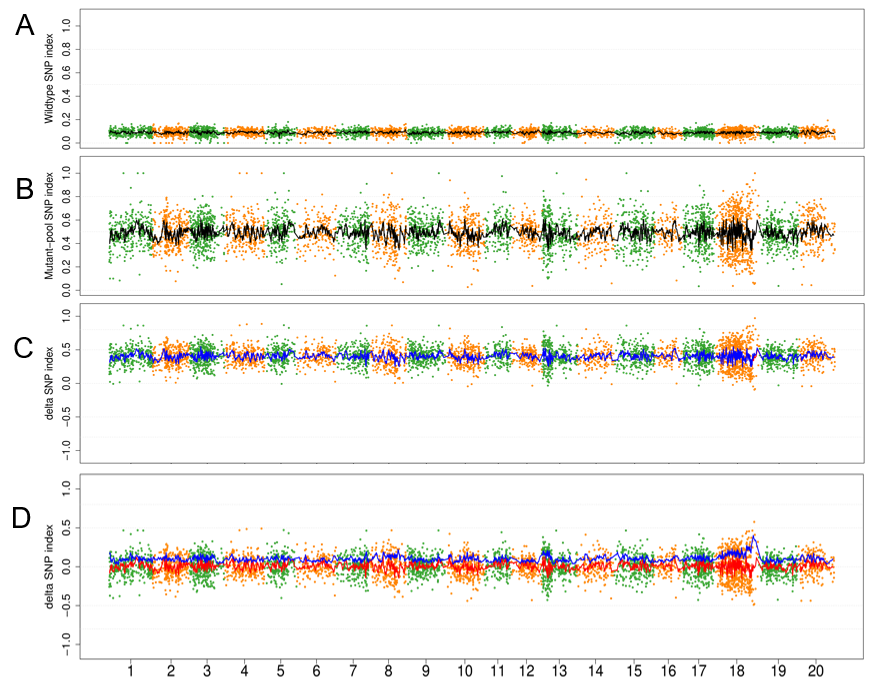


**FIGURE S6. The simulation results of various progenies in wild-type bulk of population Mut03 from non-causal mutagenic cells.**  (8) 80% progenies derived from non-causal mutagenic cells**.** The rest as same as the simulation of 10% progenies.


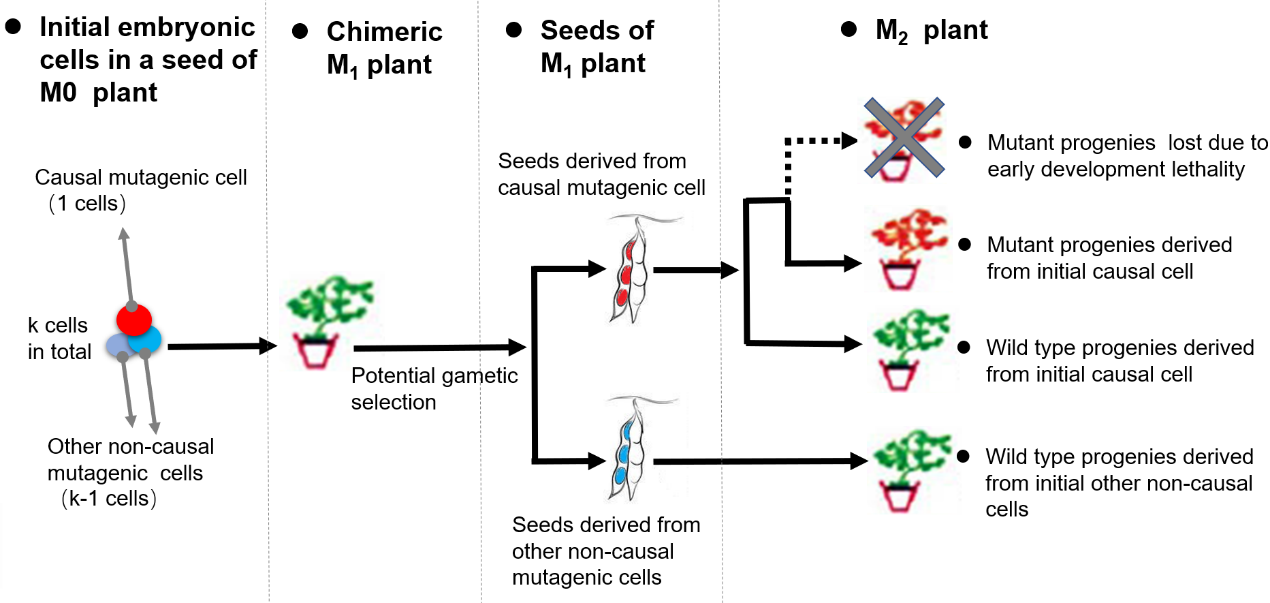


**FIGURE S7. The potential factors affecting the phenotypic differentiation ratio in M2 population**

Table S1. Summary of sequencing data

| **Bulk ID** | **Clean Reads Num** | **Clean data (Gb)** | **Average Depth** |
| --- | --- | --- | --- |
| M01-M | 306,539,448 | 45.98 | 41.80 |
| M01-W | 232,623,300 | 34.89 | 31.72 |
| M02-M | 310,566,802 | 46.59 | 42.35 |
| M02-W | 257,403,584 | 38.61 | 35.10 |
| M03-M | 301,398,022 | 45.21 | 41.10 |
| M03-W | 249,615,764 | 37.44 | 34.04 |
| M04-M | 216,028,752 | 32.40 | 29.46 |
| M04-W | 259,078,174 | 38.86 | 35.33 |
| M05-M | 272,312,654 | 40.85 | 37.13 |
| M05-W | 261,636,138 | 39.25 | 35.68 |
| M06-M | 276,010,472 | 41.40 | 37.64 |
| M06-W | 264,436,494 | 39.67 | 36.06 |
| M07-M | 270,785,934 | 40.62 | 36.93 |
| M07-W | 262,830,512 | 39.42 | 35.84 |
| M08-M | 280,575,158 | 42.09 | 38.26 |
| M08-W | 295,919,344 | 44.39 | 40.35 |
| M09-M | 298,540,038 | 44.78 | 40.71 |
| M09-W | 250,728,212 | 37.61 | 34.19 |
| M10-M | 267,410,364 | 40.11 | 36.47 |
| M10-W | 264,235,644 | 39.64 | 36.03 |

**Table S2. Number of genomic variations during each filtering step**


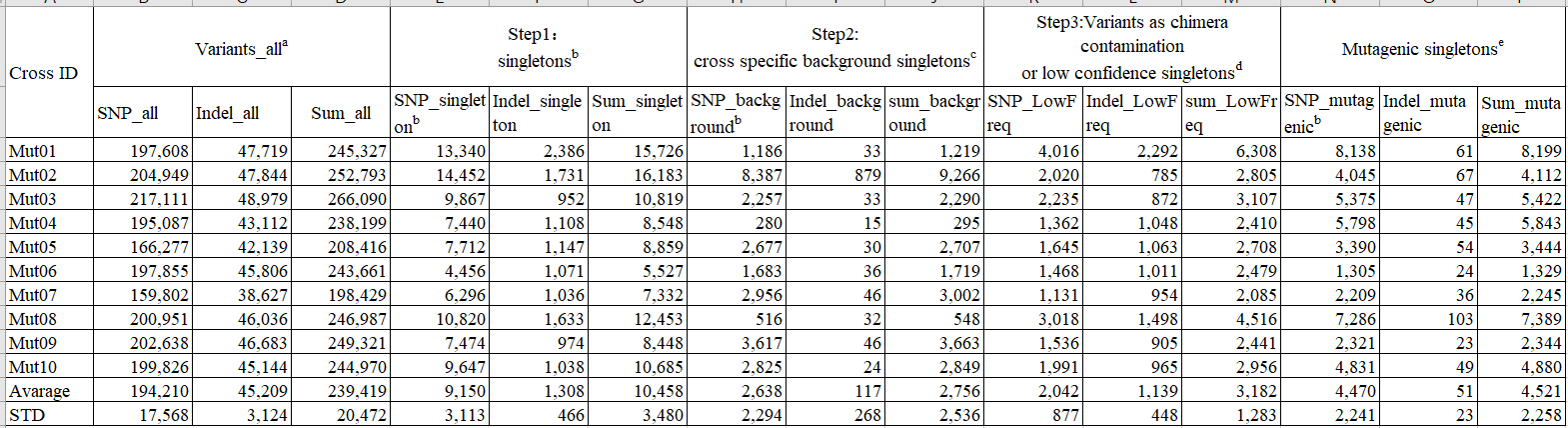


a) Only variants with reads coverage ≥ 6 could be included in this statistic.

b) Singleton variant means the variants were only detected in one cross.

c) In each cross, the singteton variants with SNP index great than 0.7 in both bulks were regarded as cross specific background singletons.

d) In each cross, the singteton variants with SNP index less than 0.3 in both bulks were regarded as low confidence singletons, any variants with SNP index=0 in mutant bulks were also eliminated as they are potential variants from chimera contamination .

e) In each cross, the remined variants were regarded as mutagenic singletons.

**Table S3. The candidate regions of M2 populations and number of mutations detected in candidate region**

| ID | Chromosome | Target region | Region size | Number of mutation in target region | Number of mutation in coding region | Number of candidate causal mutation* |
| --- | --- | --- | --- | --- | --- | --- |
|
| Mut01 | Chr08 | 10~18Mb | 8Mb | 68 | 16 | 4 |
| Mut02 | Chr07 | 4~8Mb | 4Mb | 42 | 8 | 3 |
| Mut03 | Chr18 | 50~55Mb | 5Mb | 31 | 16 | 0 |
| Mut04 | Chr09 | 1~12.5Mb | 11.5Mb | 71 | 12 | 1 |
| Mut05 | Chr20 | 40~45Mb | 5Mb | 31 | 6 | 2 |
| Mut06 | Chr14 | 1~4Mb | 3Mb | 6 | 4 | 1 |
| Mut07 | Chr10 | 45~52Mb | 7Mb | 47 | 13 | 2 |
| Mut08 | Chr16 | 5~28M | 23Mb | 209 | 35 | 3 |
| Mut09 | Chr12 | 0~5Mb | 5Mb | 28 | 18 | 1 |
| Mut10 | Chr07 | 40~45Mb | 5Mb | 40 | 23 | 2 |

*Candidate causal mutation should meet two criteria: 1) it can change the amino acid sequence of protein; 2) mutation frequency:SNP index = 1 in the mutant bulk.

**Table S4. Mutation information in the candidate region of Mut01 population**


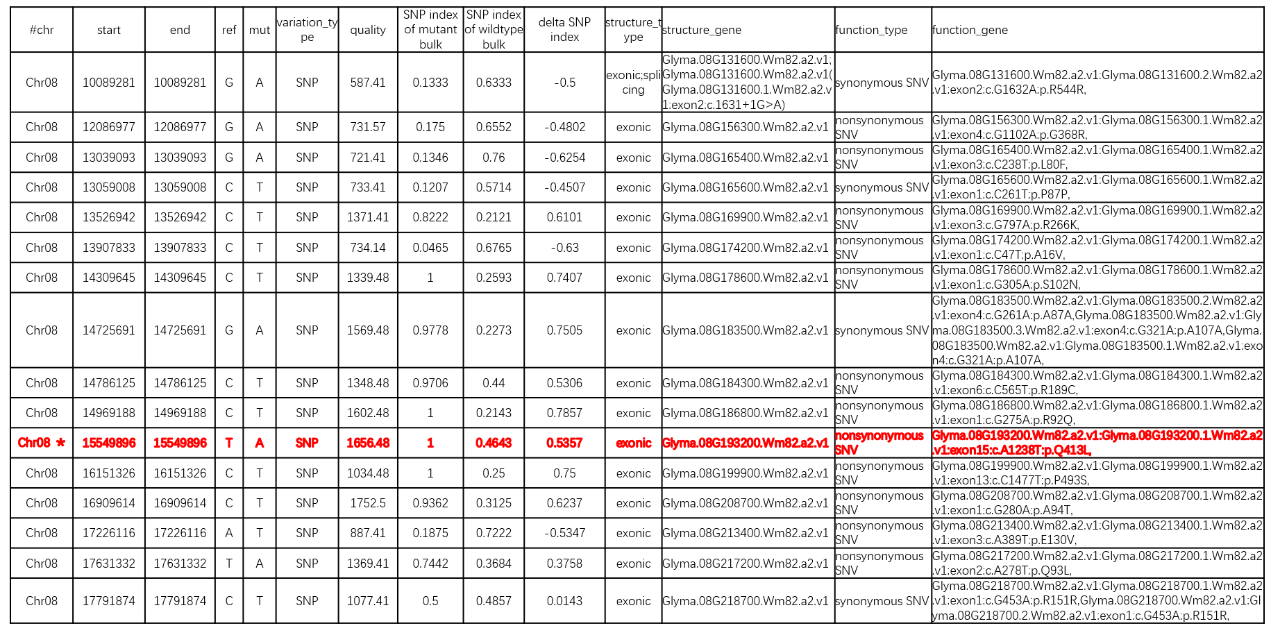


* I­­­­­ndicates the causal mutation

**Table S5. Mutation information in the candidate region of Mut07 population**


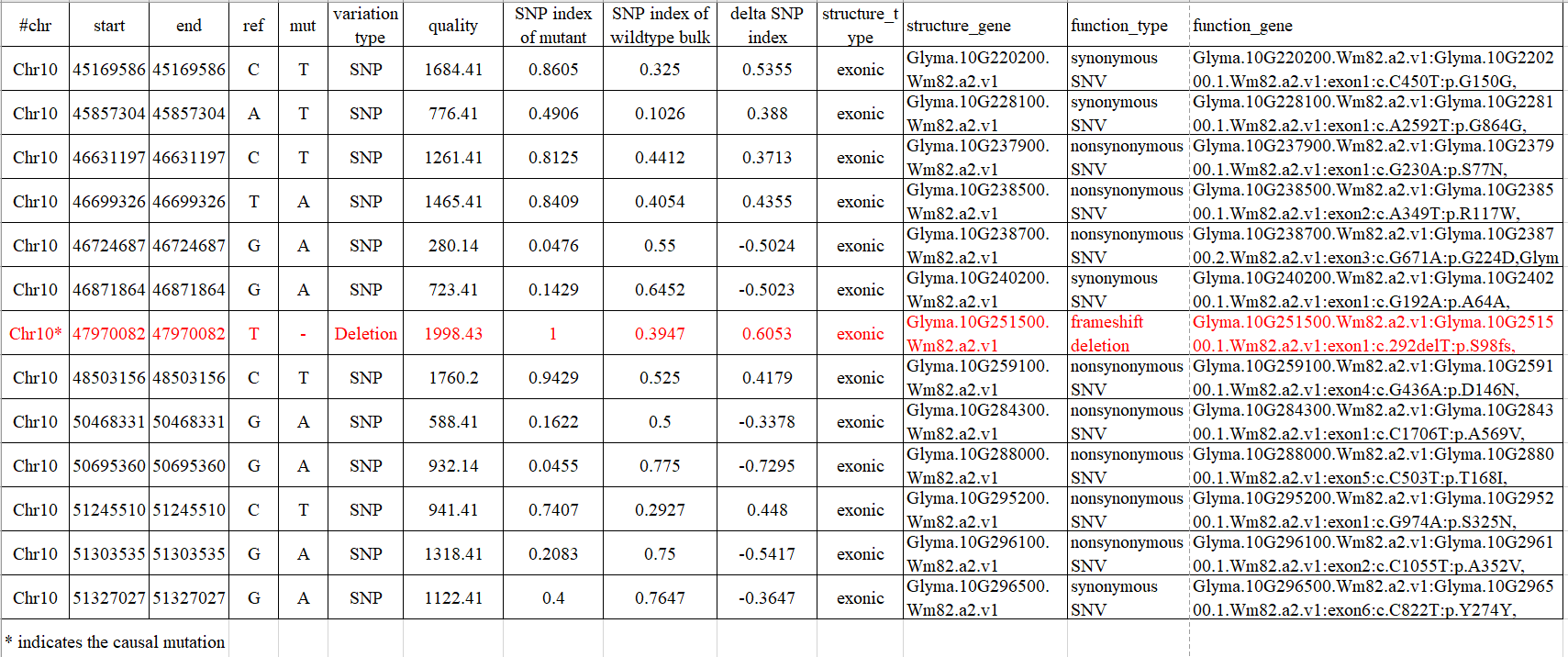


* Indicates the causal mutation

**Table S6. Number of variations retained as mutagenic mutations without step 1**

| Cross ID | SNP | Indel | Sum |
| --- | --- | --- | --- |
| Mut01 | 87,689 | 9,886 | 97,575 |
| Mut02 | 84,879 | 10,054 | 94,933 |
| Mut03 | 86,568 | 10,092 | 96,660 |
| Mut04 | 83,953 | 9,173 | 93,126 |
| Mut05 | 80,656 | 9,512 | 90,168 |
| Mut06 | 79,724 | 9,243 | 88,967 |
| Mut07 | 76,706 | 8,825 | 85,531 |
| Mut08 | 90,739 | 10,867 | 101,606 |
| Mut09 | 80,917 | 9,442 | 90,359 |
| Mut10 | 82,520 | 9,320 | 91,840 |
| Avarage | 83,435 | 9,641 | 93,077 |
| STD | 4,184 | 591 | 4,720 |
